# Fruit respiration: putting alternative pathways into perspective

**DOI:** 10.1111/nph.70882

**Published:** 2026-02-10

**Authors:** Ariadna Iglesias‐Sanchez, Sergio García‐Carbonell, Alisdair R. Fernie, Marta Pujol, Igor Florez‐Sarasa

**Affiliations:** ^1^ Centre for Research in Agricultural Genomics (CRAG) CSIC‐IRTA‐UAB‐UB Edifici CRAG Campus UAB, Bellaterra 08193 Barcelona Spain; ^2^ Max‐Planck‐Institut für Molekulare Pflanzenphysiologie Am Mühlenberg 1 14476 Potsdam‐Golm Germany; ^3^ Institut de Recerca i Tecnologia Agroalimentàries (IRTA) Edifici CRAG Campus UAB, Bellaterra 08193 Barcelona Spain

**Keywords:** alternative respiratory pathways, climacteric ripening, ethylene, fruit growth, fruit respiration, secondary metabolism

## Abstract

Over the past century, research has significantly advanced our understanding of fruit respiration, from (eco)physiological processes to molecular mechanisms. This review focuses on the functional relevance and regulatory roles of mitochondrial alternative respiratory pathways (ARPs) during fruit growth and ripening. We revisit classical distinctions between climacteric and nonclimacteric fruits, considering recent insights into the alternative oxidase, uncoupling proteins, and type II NAD(P)H dehydrogenases (NDIIs). These components are increasingly recognized as central to maintaining metabolic flexibility, energy balance, and redox homeostasis, supporting both primary and secondary metabolism. We highlight how CO_2_ refixation and organic acid metabolism, often displaying C_4_/CAM‐like features, impose specific demands on mitochondrial electron transport, and how spatial heterogeneity in metabolism and O_2_ availability across fruit tissues can shape respiratory activity. Interactions between fruit photosynthesis and respiration remain poorly understood, particularly under stress. The interplay between respiration, ethylene biosynthesis, and signaling is discussed, emphasizing feedback loops involving mitochondrial retrograde regulation and redox‐sensitive control of ripening. Key knowledge gaps include *in vivo* flux analyses, tissue‐resolved energy profiling, and functional characterization of underexplored ARP components. Finally, we outline postharvest and metabolic engineering strategies targeting ARPs as complementary to ethylene‐centered approaches to improve fruit quality, stress resilience, and nutritional value.

**Table:** 

	Content	
	Summary	54
I.	Introduction	55
II.	Fruit respiration during growth	55
III.	Fruit respiration and photosynthetic carbon assimilation	57
IV.	Fruit respiration, CO_2_ refixation, and organic acid metabolism	57
V.	Fruit respiration and the mitochondrial electron transport chain	58
VI.	Climacteric and nonclimacteric fruit ripening	60
VII.	Ethylene biosynthesis and regulation in current fruit models	60
VIII.	Ethylene and respiration during climacteric ripening: a chicken‐egg situation?	62
IX.	The relationships between climacteric and alternative respiration	64
X.	Respiration and secondary metabolism in fruits	65
XI.	Conclusions and future perspectives	67
	Acknowledgements	68
	References	69

## I. Introduction

At the beginning of the last century, research on fruit ripening and postharvest physiology focused primarily on respiration, with little consideration of ethylene, as documented in the classic work of Kidd, West and colleagues (reviewed by Laties, 1995). It was only later that ethylene was identified as the key gaseous hormone responsible for triggering ripening in fruits (Gane, 1934). This discovery shifted research toward understanding the synthesis, perception, and action of ethylene, which, together with respiration, became defining traits for distinguishing climacteric from nonclimacteric fruits (Barry & Giovannoni, 2007; Paul *et al*., 2012; Zenoni *et al*., 2023). Numerous reviews have addressed biochemistry and metabolism of fleshy fruits (i.e. Biale & Young, 1981; Tucker, 1993; Carrari & Fernie, 2006; Sweetman *et al*., 2009; Beauvoit *et al*., 2018; Walker & Famiani, 2018; Pott *et al*., 2019; Zhu *et al*., 2022), whereas other fruit types remain comparatively less studied (Seymour *et al*., 2013). Among these, reviews focused on the nature and role of respiration were mostly published in the last century (i.e. Biale & Young, 1981; Tucker, 1993), with only a few recently addressing mitochondrial metabolism during fruit ripening (Perotti *et al*., 2014; Hewitt & Dhingra, 2020). In this review, we provide an overview of respiratory metabolism in fleshy fruits during fruit development, from growth to ripening, with special emphasis on recent advances in the role and regulation of alternative respiratory pathways (ARPs). We also identify key questions that will guide future research. That said, current evidence already highlights the pivotal role of the ARPs in providing metabolic flexibility, enabling fruits to meet energy and carbon demands from primary and secondary metabolic pathways throughout development and ripening.

**Fig. 1:**
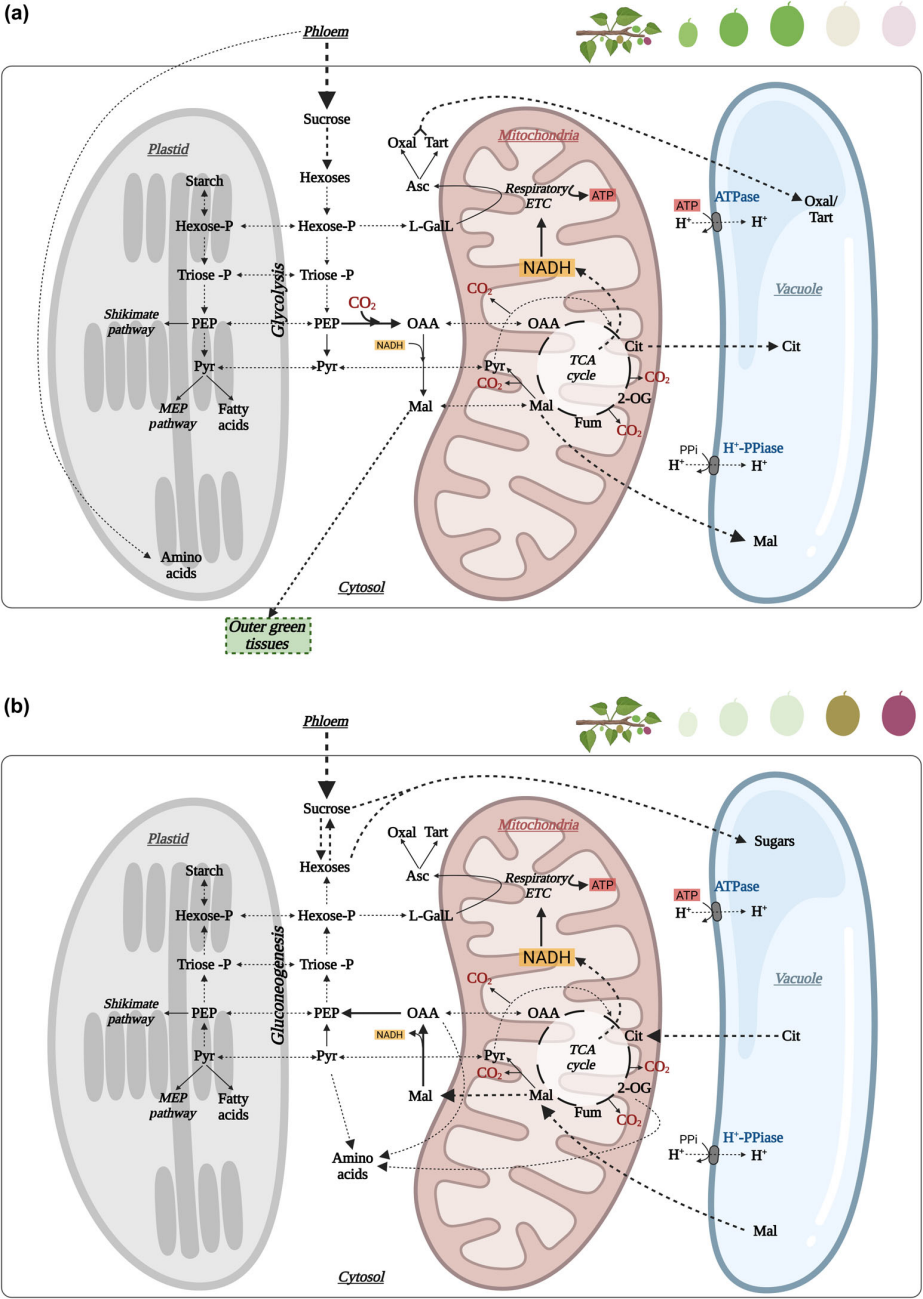
Primary carbon metabolism and intracellular compartmentation during early fruit development (a) and ripening (b). Schematic overview of the interactions among plastids, mitochondria, vacuoles, and the cytosol, emphasizing major carbon fluxes, storage processes, and metabolic shifts across developmental stages. Sucrose imported via the phloem represents the dominant carbon source for fruit metabolism, especially in inner, heterotrophic tissues. (a) Cell division/expansion stages. Glycolysis provides phosphoenolpyruvate (PEP) and pyruvate, the latter entering mitochondria where it fuels the TCA cycle and NADH production to support ATP synthesis via the mitochondrial electron transport chain (ETC). PEP carboxylase (PEPC) refixes mitochondrial CO_2_ into oxaloacetate, which is subsequently reduced to malate by malate dehydrogenase (MDH). Citrate and malate accumulate in the vacuole through the action of H^+^‐ATPases and H^+^‐pyrophosphatases, which also contribute to vacuolar acidification. Malate produced in inner (heterotrophic) tissues can be transported toward outer, green tissues, where its decarboxylation by NADP‐malic enzyme (NADP‐ME) increases chloroplastic CO_2_ availability for Rubisco (see Fig. 2). Ascorbate (Asc) biosynthesis and related organic acid pathways (notably tartaric acid in grapes and oxalate in berries) are also highly active during early development. (b) Ripening stages. Vacuolar organic acids are progressively remobilized and can be channeled through gluconeogenesis to generate triose‐P, hexose‐P, and hexoses, which either accumulate in the vacuole (e.g. glucose, fructose) or contribute to the synthesis of amino acids and downstream secondary metabolites (see Fig. 5). These metabolic rearrangements accompany the transition from an acid‐dominated to a sugar‐dominated fruit profile during ripening. This figure was created in BioRender, Pujol, M. (2026) (https://BioRender.com/2viyx3q).

**Fig. 2:**
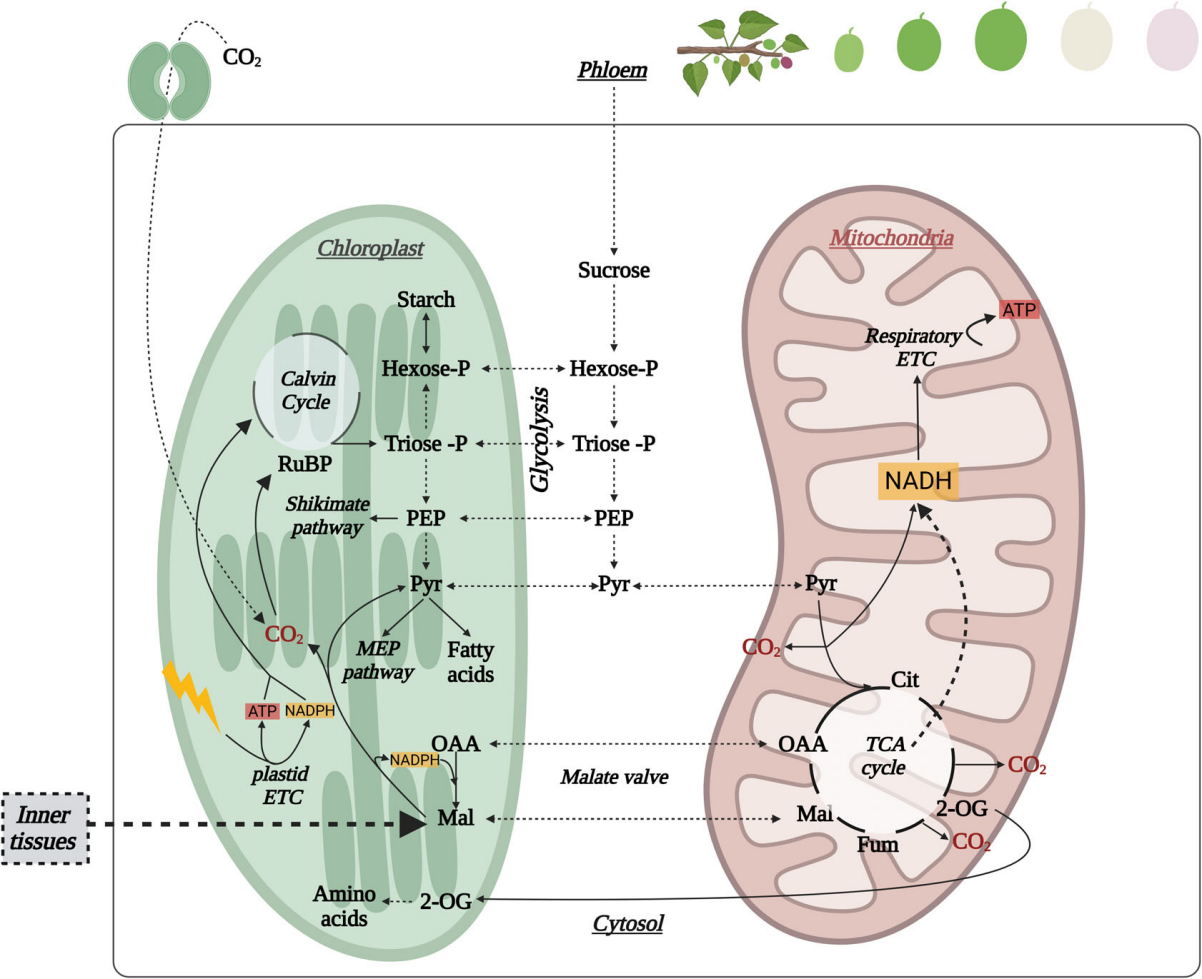
Chloroplast–mitochondria metabolic interactions during early fruit development. Schematic representation of carbon and redox exchange between chloroplasts and mitochondria in photosynthetically active outer fruit tissues. CO_2_ released from malate decarboxylation (imported from inner tissues) or from other decarboxylating reactions (see main text) is reassimilated in the chloroplast by Rubisco, supported by ATP and NADPH generated through the plastid electron transport chain. When light availability exceeds chloroplastic reductant demand, excess reducing power is exported to the cytosol and mitochondria via the malate valve. Triose phosphates (TPs) produced in the Calvin cycle provide precursors for starch, sucrose, fatty acids, and amino acid biosynthesis. Chloroplastic TPs and other glycolytic intermediates can also be exported to the cytosol to fuel respiratory metabolism. Pyruvate (Pyr), oxaloacetate (OAA), and malate (Mal) constitute key exchange metabolites linking plastidial and mitochondrial pathways. In mitochondria, pyruvate and organic acids are oxidized through the TCA cycle, generating NADH that feeds the mitochondrial electron transport chain to produce ATP. Exchange of citrate (Cit), malate, oxaloacetate, and 2‐oxoglutarate (2‐OG) across compartments helps balance carbon skeleton supply and redox status. Overall, these interorganellar fluxes provide metabolic flexibility and support energy and redox homeostasis during early fruit growth and environmental fluctuations. This figure was created in BioRender, Pujol M. (2026) (https://BioRender.com/edx0i2v).

**Fig. 3:**
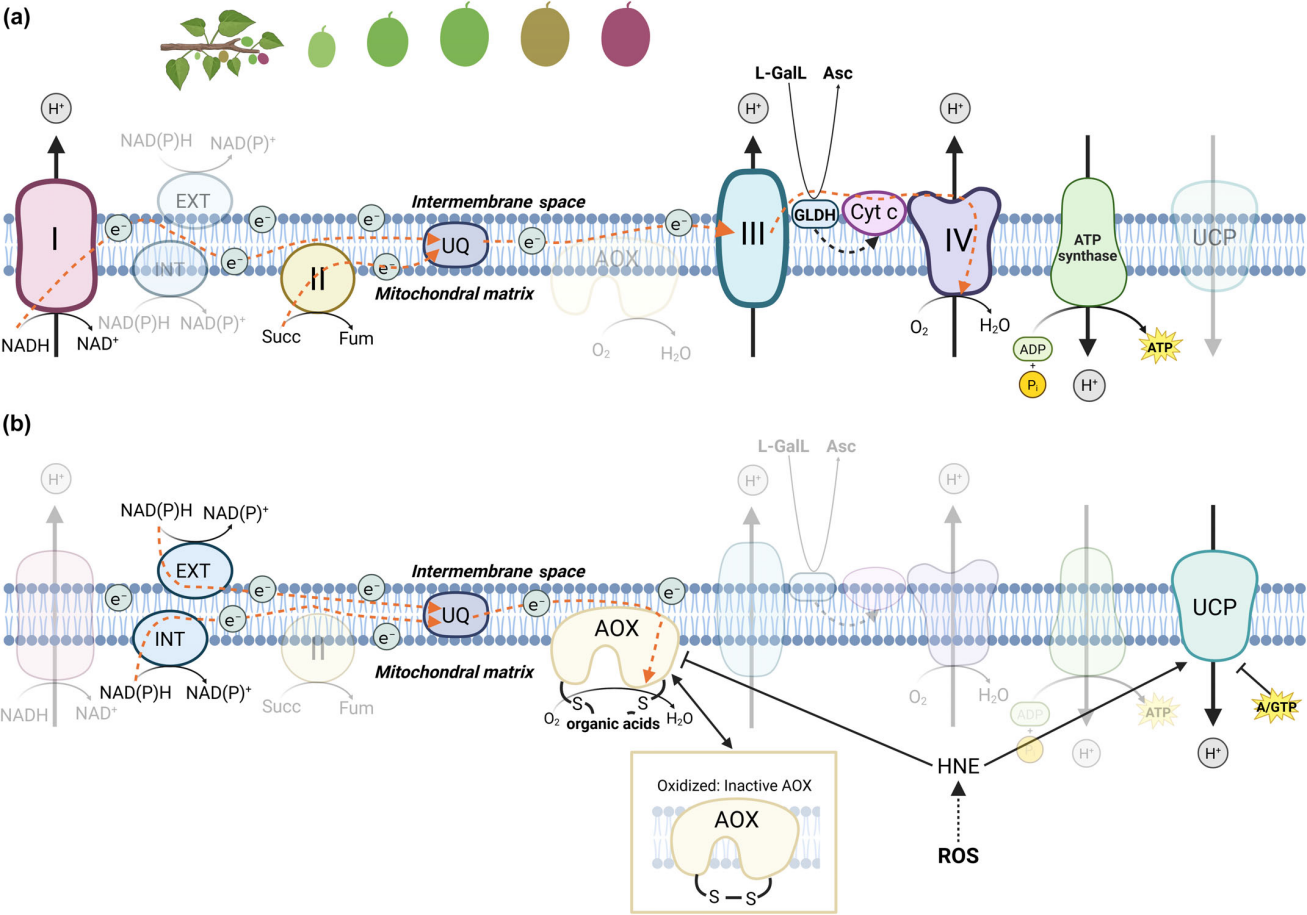
Plant mitochondrial electron transport chain (mETC) showing (a) the canonical respiratory pathway for oxidative phosphorylation and (b) the nonphosphorylating alternative respiratory pathways (ARPs). (a) Electrons from NAD(P)H and FADH_2_ are transferred through four major multisubunit complexes: Complex I (NADH dehydrogenase), Complex II (succinate dehydrogenase), Complex III (cytochrome *bc*_*1*_ complex), and Complex IV (cytochrome *c* oxidase, COX). As electrons flow through Complexes I, III, and IV, protons are translocated from the mitochondrial matrix to the intermembrane space, generating an electrochemical proton gradient. This gradient through proton motive force drives ATP synthesis by Complex V (ATP synthase), which catalyzes the formation of ATP from ADP and inorganic phosphate (P_i_). This system of electron‐transport‐coupled ATP synthesis enables a tight regulation or ‘respiratory control’ of the electron transport chain by ADP and P_i_ availability and, therefore, by the energy demand (i.e. ATP : ADP ratio) of the cell. (b) ARPs consist of several alternative proteins including the type II NAD(P)H dehydrogenases, alternative oxidases (AOXs), and uncoupling proteins (UCPs), which can bypass parts of the canonical chain. The AOX pathway allows direct electron transfer from ubiquinol to molecular oxygen (O_2_), reducing it to water (H_2_O) without contributing to proton gradient formation. AOX bypasses the proton‐pumping activities of Complexes III and IV. While some ATP can still be generated via Complex I, electron transport from alternative NAD(P)H dehydrogenases or Complex II to AOX is entirely uncoupled from ATP synthesis. The AOX is encoded by a multigene family containing AOX1‐ and AOX2‐type genes, some of those displaying distinct organ and developmental patterns of expression (Costa *et al*., 2014). Most plant AOXs have been found in a dimeric state, which can be redox‐regulated through a disulfide/sulfhydryl system, with some exceptions. Once its disulfide bond is reduced, AOX activity can be stimulated through allosteric regulation by interaction with organic acids, such as pyruvate, as has been demonstrated in isolated mitochondria or other *in vitro* systems (Selinski *et al*., 2018). Nevertheless, the extent to which the AOX is allosterically activated *in vivo* remains uncertain (Del‐Saz *et al*., 2018) and may depend on isoform‐specific activation properties and the coexistence of AOX homo‐ and heterodimers (McDonald, 2023). On the other hand, uncoupling proteins (UCPs) can dissipate proton gradient by facilitating proton re‐entry into the matrix, thereby uncoupling electron transport from ATP even when COX pathway is active. Previously, a UCP subfamily was identified containing six members in *Arabidopsis thaliana* (UCP1‐6), although later, most of the members were renamed as dicarboxylate carriers (UCP4‐6) (Monné *et al*., 2018). UCPs are directly inhibited by purine nucleotides (ATP, GTP, or GDP) and are activated by free fatty acids or lipid peroxidation products such as 4‐hydroxy‐2‐nonenal (HNE), the latter being strong AOX inhibitors (Barreto *et al*., 2020). This *in vitro* evidence supports a model in which AOX and UCP act sequentially under rising mitochondrial ROS levels; however, *in vivo* evidence confirming this AOX/UCP coordination, and identifying the physiological contexts in which it operates, such as fruit ripening, remains to be established. This figure was created in BioRender, Pujol M. (2026) (https://BioRender.com/bggvis3).

Plant respiration is a fundamental metabolic process that oxidizes reduced carbon compounds to CO_2_ and H_2_O, releasing energy that is conserved in ATP and reducing equivalents. Beyond ATP production and supply of carbon skeletons, respiration maintains redox balance, a function particularly critical because of the sessile lifestyle of plants (O'Leary *et al*., 2019). Furthermore, under certain conditions, respiration can generate substantial heat in reproductive tissues, providing additional physiological and evolutionary benefits (Barreto *et al*., 2025). These diverse functions require distinct respiratory flux modes to meet specific demands of the plant cells and organs (O'Leary *et al*., 2019; Sweetlove *et al*., 2025). Fruits exhibit a particularly complex metabolism due to their capacity to synthesize and accumulate a wide range of compounds related to stress responses and seed dispersal, including terpenoids, volatiles, and phenylpropanoids (Pott *et al*., 2019). Moreover, CO_2_ refixation and organic acid metabolism show C_4_/CAM‐like features (Walker & Famiani, 2018; Garrido *et al*., 2023). Fruit growth and metabolite biosynthesis rely on ATP and carbon precursors including sugars, organic acids, and amino acids, whose levels are tightly regulated by respiratory metabolism (Fig. 1). Cytosolic glycolysis, the mitochondrial TCA cycle, and the electron transport chain (Fig. 1) display marked metabolic flexibility through the operation of alternative routes and energy bypass pathways (Figs 1, 2, 3). Although the components and pathways of plant respiratory metabolism are well characterized, their regulation is still not fully elucidated (O'Leary *et al*., 2019; Le & Millar, 2023; Zhang & Fernie, 2023; Zheng *et al*., 2025), particularly within the context of complex fruit metabolic networks.

## II. Fruit respiration during growth

Pioneering studies in apple fruits established that elevated respiration during early fruit development accompanies intense cell division and expansion, with respiration rates subsequently declining as growth slows (Laties, 1995). These developmental phases are common across fleshy fruits, albeit growth rate patterns vary among species (Roch *et al*., 2020). Despite such differences, Roch *et al*. (2020) found consistent correlations between fruit growth rates and the accumulation of structural components, including lipids, cell wall components, and proteins. Building on this framework, Colombié *et al*. (2023) used flux balance modeling to reveal the pivotal role of nitrogen metabolism in determining fruit growth. Their study identified a complex trade‐off, where rapid growth driven by cell wall synthesis was linked to reduced accumulation of defense compounds, suggesting a prioritization of growth over defense mechanisms. Interestingly, carbon availability was not a limiting factor, underscoring nitrogen metabolism as a key determinant of growth capacity. In these studies, the use of different metabolic modeling approaches has therefore proven powerful for exploring metabolic determinants driving fruit growth and development (Beauvoit *et al*., 2018; Colombié *et al*., 2023), allowing researchers to circumvent the experimental challenges of measuring *in vivo* metabolic fluxes. However, such models often assume higher ATP yields of plant respiration than occur *in planta*, as they rarely account for the engagement of energy bypass systems within the mitochondrial electron transport chain (Amthor, 2023). In this context, refined manipulation of the non‐phosphorylating alternative oxidase (AOX) pathway has been proposed as a promising strategy for enhancing crop yield by reducing maintenance respiration and reallocating energy and carbon resources toward growth (Amthor, 2023). However, these proposals are based on data from vegetative tissues, with estimated AOX respiration contributing 10–50% of total respiration (Del‐Saz *et al*., 2018). Fruit respiration has not been explicitly considered in these analyses. Determining the *in vivo* contribution of alternative respiration during fruit growth, especially under stress conditions that elevate maintenance costs, remains essential for a complete understanding of respiratory control in developing fruits.

## III. Fruit respiration and photosynthetic carbon assimilation

Fruits predominantly act as sink tissues and are typically treated as such in constraint‐based models, with carbon assumed to originate entirely from phloem‐imported sucrose (Beauvoit *et al*., 2018). However, the contribution of fruit photosynthesis to overall carbon balance remains under debate (Simkin *et al*., 2020; Garrido *et al*., 2023). Evidence from several fleshy fruits suggests that carbon assimilation largely depends on refixation of respired CO_2_ (Fig. 1a), whereas external CO_2_ uptake through stomata is minor (Fig. 2) and strongly species‐dependent (Simkin *et al*., 2020; Garrido *et al*., 2023). For instance, avocado, mandarin, and cucumber exhibit relatively high stomatal densities on the fruit surface, suggesting potential for external CO_2_ uptake, although stomatal functionality remains unclear (Simkin *et al*., 2020). In tomato, fruit growth was unaffected by genetic impairment of fruit photosynthesis, likely due to compensatory carbon import from leaves (Lytovchenko *et al*., 2011). Nevertheless, induction of photosynthetic genes in tomato fruits under water stress suggests that fruit photosynthesis may sustain growth when leaf assimilation is compromised (Nicolas *et al*., 2022). Similarly, the contribution of fruit photosynthesis to its growth and quality appears to increase under abiotic stress in other species (Garrido *et al*., 2023).

Beyond its role in carbon balance, the interaction between respiratory and photosynthetic metabolism in fruits remains poorly understood. Mitochondrial metabolism supports photosynthesis, photorespiration, nitrogen metabolism, reductant transport, and redox balance (Igamberdiev & Bykova, 2023; Fig. 2), particularly in leaves under suboptimal conditions (Vanlerberghe *et al*., 2020). The TCA cycle often operates in a noncyclic mode for providing carbon precursors and reducing power for nitrogen metabolism and amino acid synthesis (Igamberdiev & Bykova, 2023; Fig. 2). While extensive studies have characterized TCA cycle enzyme functions in illuminated leaves and in roots (Zhang & Fernie, 2023), less is known about its role in fruits. The exception to this is the relevant effect of reducing the expression of mitochondrial MDH and fumarase on fruit metabolism and ripening (Centeno *et al*., 2011). Notably, the role of organic acids in fruit development was suggested in early studies combining metabolite and transcript profiles in tomato (Carrari & Fernie, 2006), and the specific function of malate was later highlighted through manipulation of the PEP carboxykinase (PEPCK) and plastidic NADP‐ME enzymes (Osorio *et al*., 2013). Although these studies primarily addressed metabolic control during ripening, they underscore that the regulation and role of the TCA cycle and organic acid metabolism during fruit growth phases remain important open questions.

## IV. Fruit respiration, CO_2_ refixation, and organic acid metabolism

Carbon flux analyses revealed an important contribution of cucumber photosynthesis to fruit growth, with limited external CO_2_ fixation but extensive internal refixation driven by high respiratory activity (Sui *et al*., 2017). This refixation is mainly mediated by phosphoenolpyruvate carboxylase enzyme (PEPC; Fig. 1a), as demonstrated in cucumber, tomato, and apple (Guillet *et al*., 2002; Sui *et al*., 2017; Garrido *et al*., 2023). A C_4_/CAM‐type mechanism operates in fruits, in which cytosolic malate generated by PEPC and malate dehydrogenase (MDH) is transported into chloroplasts from green tissues and decarboxylated by the NADP‐malic enzyme (ME) to release CO_2_ for Rubisco (Garrido *et al*., 2023; Figs 1a, 2). NADP‐ME also promotes carbon flux into soluble sugars and starch in cucumber fruits (Shan *et al*., 2023). High PEPC activity parallels malate and citrate accumulation in vacuoles (Fig. 1a), which, together with ion transport (Etienne *et al*., 2013), supports cell expansion and rapid fruit growth (Guillet *et al*., 2002; Sui *et al*., 2017).

Recent syntheses highlight that the direction of flux through malate‐linked reactions shifts dynamically across fruit development (Fig. 1). During growth, PEPC, MDH, and NADP‐ME primarily support anaplerosis and CO_2_ refixation (Fig. 1b), whereas during ripening organic acids released from the vacuole can be recycled into sugars and amino acid skeletons through gluconeogenesis (Fig. 1b). According to Walker *et al*. (2021), both the PEPCK‐ and PPDK‐dependent pathways operate in fruits, and their relative contribution depends on species, developmental stage, and N status. Vacuolar storage and release of malate and citrate are therefore crucial for coordinating catabolic and gluconeogenic fluxes, with PEPCK, MDH, ME, and PPDK forming a regulatory hub that links cytosolic pH, respiration, and sugar metabolism during fruit growth and ripening (Fig. 1).

Other organic acids, notably tartaric and oxalic acids, also accumulate in vacuoles during early development (Fig. 1a). In grape, tartaric acid, derived from ascorbate intermediates, acts as an osmotic regulator and carbon sink, while also contributing to redox and apoplastic signaling during ripening (Walker & Famiani, 2018; Burbidge *et al*., 2021). Oxalic acid may also assist in ionic balance and calcium storage, reinforcing the multifunctional nature of organic acids in fruit development (Walker & Famiani, 2018).

Beyond these structural functions, organic acids exert regulatory and signaling roles. Coordinated activity of PEPC, MDH, ME, citrate synthase, and aconitase integrates organic acid turnover with sugar metabolism and respiration (Batista‐Silva *et al*., 2018; Fig. 1). These pathways respond to hormonal and environmental cues, with ABA and ethylene modulating malate and citrate levels during ripening and stress. Moreover, malate, citrate, and 2‐oxoglutarate act as metabolic signals influencing redox balance and carbon–nitrogen interactions, underscoring their central role in regulating fruit growth, ripening, and quality (Batista‐Silva *et al*., 2018). Indeed, this link was underscored by an early multi‐omics study across tomato fruit ripening which revealed canonical ripening differentially expressed genes were highly correlated to differences in the levels of TCA cycle intermediates (Carrari *et al*., 2006). This observation was mechanistically confirmed in subsequent transgenic experiments including those mentioned in Section III (Centeno *et al*., 2011; Osorio *et al*., 2013) as well as a more recent study in which the bacterial‐type PEPC was subject to gene editing (Martinez Rivas *et al*., 2025).

Consistent with this integrative view, spatial heterogeneity in metabolism suggests that CO_2_ refixation, organic‐acid turnover, and sugar metabolism vary across fruit tissues (pericarp, locule, and vascular regions), reflecting distinct coordination between photosynthetic competence, respiratory activity, and development. Metabolomic analyses in tomato reveal marked differences in organic acid and sugar profiles between outer and inner tissues (Lemaire‐Chamley *et al*., 2019), while modelling and mechanistic studies support internal CO_2_ refixation and malate‐mediated shuttles coupling photosynthetic and respiratory processes across tissue layers (Garrido *et al*., 2023). Such compartmentalized fluxes likely optimize the internal carbon economy and redox homeostasis of developing fruits, suggesting that distinct tissues could differentially engage ARPs within the mitochondrial electron transport chain (mETC) to meet their specific metabolic demands.

## V. Fruit respiration and the mitochondrial electron transport chain

The mETC is essential for energy production in plants through oxidative phosphorylation at the inner mitochondrial membrane (Fig. 3). In parallel, the plant mETC is characterized by its ARPs, including the internal and external type II NAD(P)H dehydrogenases (NDIIs), uncoupling proteins (UCPs), and AOXs (Fig. 3). Although these pathways are not coupled with ATP synthesis, substantial evidence supports their important roles in providing cell metabolic flexibility while avoiding the formation of reactive oxygen (ROS) and nitrogen (RNS) species (Del‐Saz *et al*., 2018; Barreto *et al*., 2020; Fig. 3). UCPs dissipate the proton gradient, thereby uncoupling electron transport from ATP synthesis (Barreto *et al*., 2020; Fig. 3). *In vitro* studies have proposed additional roles for Arabidopsis UCP1 and 2 as metabolite transporters, potentially facilitating redox‐metabolite exchanges between cellular compartments during photorespiration (Monné *et al*., 2018). While the study of Sweetlove *et al*. (2006) provided *in vivo* evidence for a role of UCP1 in photorespiration, the relative importance of its dual operation, as uncoupler vs metabolite transporters remains unresolved (Barreto *et al*., 2020). This question is particularly unexplored in fruits. On the other hand, the NDIIs and AOXs enable electron transport without generating a proton gradient (Fig. 3). Several NDIIs transfer electrons from NAD(P)H to the ubiquinone (UQ) pool, bypassing proton‐pumping across Complex I. These include internal NDIIs on the matrix side of the inner mitochondrial membrane, and external NDIIs on the intermembrane space side of the inner membrane (Rasmusson *et al*., 2020; Fig. 3). While their nature and regulation are well characterized in Arabidopsis (Rasmusson *et al*., 2020; Jethva *et al*., 2023), their roles in fleshy fruits remain largely unknown. Finally, the AOX protein is inserted in the inner membrane of plant mitochondria and branches the cytochrome *c* oxidase (COX) pathway at the level of the UQ pool, bypassing proton translocation at Complexes III and IV (Fig. 3). In general, the ARPs have gained attention as potential targets for improving crop performance under abiotic and biotic stress, yet their roles in nonphotosynthetic sink tissues such as fruits remain insufficiently studied (Del‐Saz *et al*., 2018; McDonald, 2023). Furthermore, the mETC contributes to photosynthetic performance by acting as a sink for excess of reducing power from chloroplasts under high light or ammonium stress (Rasmusson *et al*., 2020; Vanlerberghe *et al*., 2020). Chlorophyll fluorescence measurements in the fruits of several species indicate a functional electron transport chain during early fruit development (Simkin *et al*., 2020; Garrido *et al*., 2023; Fig. 2), which responds to high light intensities in grapes (Garrido *et al*., 2023). Under such conditions, dynamic activation of ARPs enhances metabolic flexibility, balancing carbon and energy supply and demand (Del‐Saz *et al*., 2018; O'Leary *et al*., 2019). Although the role of ARPs in fruit stress responses remains poorly understood, it is reasonable to hypothesize that these pathways, well characterized in leaves, also play important roles in fruit primary metabolism.

Another important role of mitochondria in maintaining cellular redox balance lies in the involvement of the mETC in the synthesis of ascorbate (Matos *et al*., 2022). The final step of ascorbate synthesis is catalyzed by the L‐galactono‐1,4‐lactone dehydrogenase (L‐GLDH), located at the inner membrane and using oxidized cytochrome *c* as an electron acceptor (Fig. 3). Matos *et al*. (2022) have proposed that AOX activity may influence ascorbate synthesis by modulating the redox state of the mETC, particularly under stress, though *in vivo* confirmation is lacking. Despite the importance of ascorbate in fruit metabolism (Fenech *et al*., 2019) and ripening (Arabia *et al*., 2024), this relationship has only recently been examined in isolated papaya mitochondria (Silva *et al*., 2024). Measurements of *in vivo* electron partitioning between AOX and COX pathways (Del‐Saz *et al*., 2018) in L‐GLDH‐ or ARP‐modified fruits could provide crucial insights into mitochondrial control of ascorbate synthesis. Decros *et al*. (2023) have recently found that increased ascorbate synthesis is tightly associated with growth at early developmental stages in tomato fruits, although ascorbate was found mainly in its oxidized form. A drastic shift from oxidized to reduced ascorbate coincides with the transition from cell division to expansion. These results collectively denote a strong link between growth and redox metabolism (Decros *et al*., 2023). Across diverse species, high levels of oxidized ascorbate characterize the onset of fruit development, with nonclimacteric fruits maintaining a more oxidized state throughout (Roch *et al*., 2020). Interestingly, Roch *et al*. (2020) suggested that the nature and abundance of compounds accumulated during the growth phase may influence the climacteric character of ripening. Ripening, tightly linked to respiration and ethylene synthesis, is further examined in the following sections.

## VI. Climacteric and nonclimacteric fruit ripening

The ripening process occurs during the last stages of fruit development and determines fruit quality, as numerous physiological and biochemical changes take place that affect appearance and nutritional value. These include aroma production, softening, accumulation of sugars, organic acids and vitamins, and variations in their antioxidant potential among others. Many studies have been conducted to understand the physiological, genetic, epigenetic, and metabolic factors underlying fruit ripening (Perotti *et al*., 2014; Giovannoni *et al*., 2017; Chen *et al*., 2020; Brumos, 2021; Li *et al*., 2022). Based on their ripening behavior, fleshy fruits can be divided into climacteric or nonclimacteric (Paul *et al*., 2012). In climacteric fruits such as tomato, banana, apple, pear, or avocado, ripening initiation is linked to a peak in ethylene production and a burst in respiration, which trigger major transcriptomic and metabolic reprogramming (Barry *et al*., 2000). By contrast, nonclimacteric fruits like strawberry, grapes, cherry, or citrus do not display an ethylene peak or an increase in respiration during ripening. Instead, other hormones such as ABA, brassinosteroids, or jasmonic acid orchestrate the ripening process (Perotti *et al*., 2023). Recently, this binary classification is being challenged, since there is a wide variety of fruits with different levels of ethylene production and patterns of respiration during ripening, highlighting the complexity of the process (Paul *et al*., 2012). Among these, *Cucumis melo* L. has become an alternative model to study fruit ripening since climacteric and nonclimacteric varieties coexist within the species (Pujol & Garcia‐Mas, 2023). The availability of gene‐editing tools and high‐sensitivity ethylene detection methods in melon (Pereira *et al*., 2017; Giordano *et al*., 2022), together with recent advances in O_2_ consumption measurements in fruit tissues (Iglesias‐Sanchez *et al*., 2025), now positions this species as an excellent model for dissecting the genetic and regulatory mechanisms of ripening and for refining the distinction between climacteric and nonclimacteric behaviors.

## VII. Ethylene biosynthesis and regulation in current fruit models

**Fig. 4:**
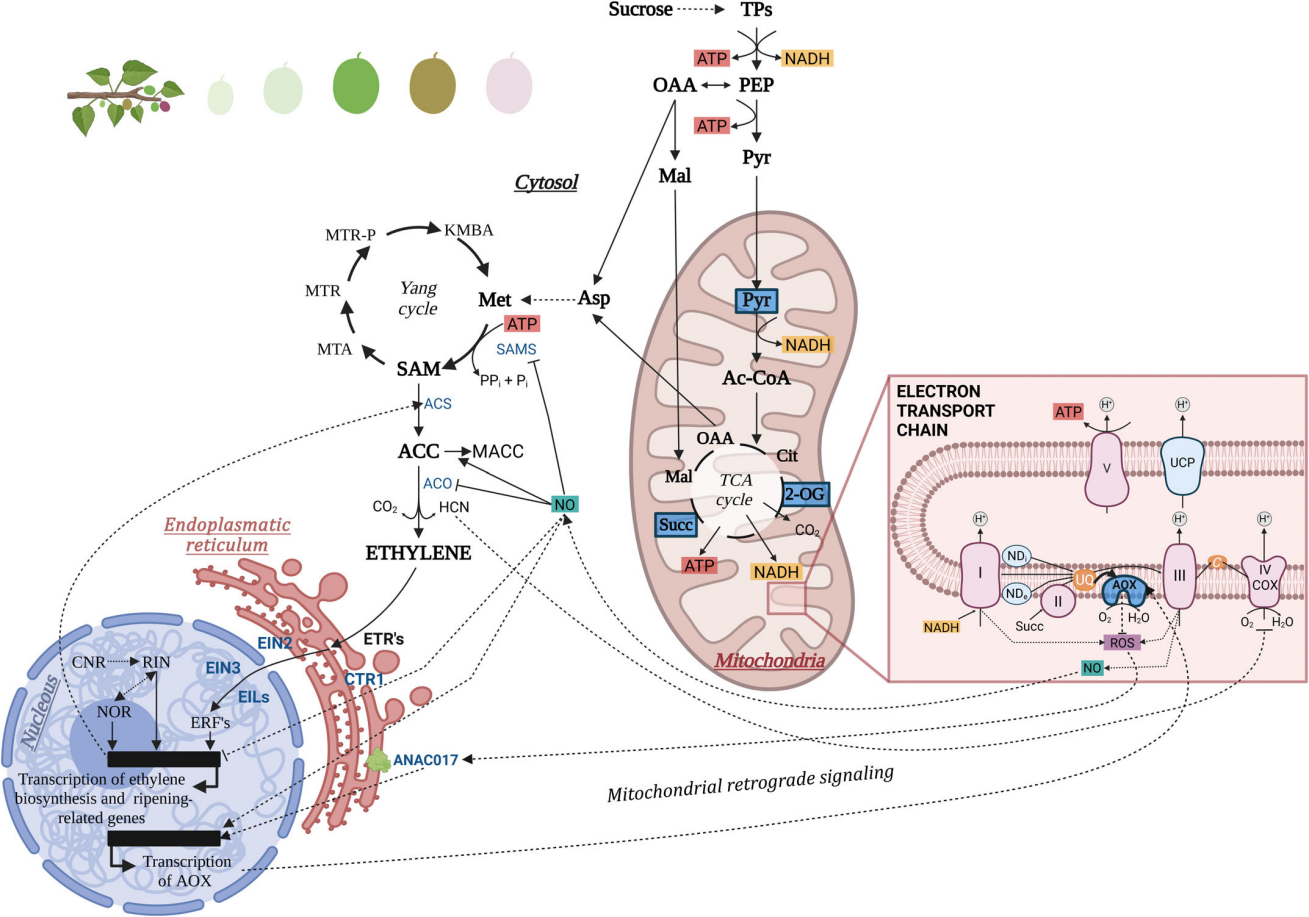
Schematic overview of the interactions between mitochondrial metabolism, ethylene biosynthesis and the main signaling pathways involved in the regulation of nuclear gene expression during the climacteric event. Cytosolic sucrose is metabolized into triose phosphates (TPs), which feed glycolysis to produce phosphoenolpyruvate (PEP), pyruvate (Pyr), and malate (Mal). These intermediates enter mitochondria, where Pyr, 2‐oxoglutarate (2‐OG), and succinate (Succ), highlighted in blue, can act as potent allosteric activators of AOX. Pyruvate is converted into acetyl‐CoA (Ac‐CoA), feeding the TCA cycle and generating ATP and substantial NADH. NADH is subsequently oxidized by the mitochondrial electron transport chain (mETC, see details at Fig. 3), which includes the alternative respiratory pathways (ARPs, shown in blue). ARPs enhance mETC turnover allowing high respiratory fluxes during climacteric ripening. The oxalacetate (OAA) produced through respiratory metabolism is converted into aspartate (Asp) which serves as the primary precursor for methionine (Met). In the ethylene biosynthetic pathway, Met and ATP are used by S‐adenosylmethionine (SAM) synthase (SAMS) to produce SAM. Then, 1‐aminocyclopropane‐1‐carboxyliase synthase (ACS) converts SAM into 1‐aminocyclopropane‐1‐carboxylic acid (ACC) and 5′‐methylthioadenosine (MTA). MTA is recycled back to Met through the Yang Cycle, producing 5′‐methylthioribose (MTR), 5′‐methylthioribose‐1‐phosphate (MTR‐P), and 2‐keto‐4‐methylthiobutyric acid (KMBA). Finally, ACC is oxidized by ACC oxidase (ACO) to generate ethylene, with the concomitant release of CO_2_ and hydrogen cyanide (HCN) byproducts. Among the ethylene biosynthetic enzymes, SAMS also participates in additional metabolic pathways (Barry & Giovannoni, 2007), whereas ACS and ACO are pivotal enzymes in ethylene production tightly regulated at transcriptional and posttranscriptional levels. Ethylene binding to Ethylene Receptors (ETRs) activates a signaling cascade involving different components (CRT1, EIN 2, EIN3 and EILs), which upregulate the Ethylene Response factors (ERFs) (see more details at the main text). ERFs finally activate genes involved in ethylene biosynthesis (i.e. ACS) and many other ripening‐related genes, all of them being also regulated by the ripening master regulators RIN, NOR, and CNR. Multiple regulatory connections exist between ethylene signaling and mitochondrial function. A major link is mitochondrial retrograde signaling (MRR), mediated by the transcription factor ANAC017 (well‐described in Arabidopsis but not in fleshy fruit species). ANAC017 is anchored into the endoplasmic reticulum (ER) membrane and is released by proteolytic cleavage upon mitochondrial dysfunction signals, including reactive oxygen species (ROS). Activated ANAC017 induces nuclear gene expression of AOX and other mitochondrial dysfunction stimulon genes. In turn, AOX activity modulates ROS and nitric oxide (NO) levels. NO inhibits ethylene biosynthesis through several post‐translational mechanisms: inhibition of SAMS (via S‐nitrosylation), stimulation of ACC conversion to malonyl‐ACC (MACC), and inhibition of ACO (via binding and chelation to form an ACC‐ACO‐NO complex). Moreover, NO transcriptionally downregulates ACS gene expression while upregulating AOX. Finally, HCN produced during ethylene synthesis may also participate in the crosstalk between ethylene and AOX signaling paths by inhibiting COX and promoting overreduction of the mETC, a condition counterbalanced by the activity of ARPs. Altogether, these interconnected pathways coordinate respiratory metabolism, redox homeostasis, ethylene synthesis, and responsiveness, thus ensuring the integrated control of climacteric fruit ripening. This figure was created in BioRender, Pujol M. (2026) (https://BioRender.com/836cdfn).

A central question in fruit biology is what triggers the onset of climacteric ripening. Ethylene acts as the primary hormone controlling this process, and its biosynthesis, perception, and signaling pathways have been extensively studied in tomato, the model species for climacteric ripening (Barry & Giovannoni, 2007; Liu *et al*., 2015). Ethylene biosynthesis starts with methionine (Fig. 4). Beyond being its precursor, methionine also participates in various physiological processes that require low but stable cellular levels (Xu & Zhang, 2015). The Yang cycle synthesizes and recycles both methionine and its derivative, S‐adenosylmethionine (SAM). During (post‐)climacteric ripening, the Yang cycle genes are highly expressed, ensuring methionine recycling from 5′‐methylthioadenosine (MTA). This coordinated regulation of the Yang cycle and ethylene biosynthesis helps control the ripening progression and delays senescence (Fig. 4). Climacteric fruits exhibit two different systems of ethylene production. System‐1 maintains basal, self‐inhibitory ethylene levels during fruit growth in both climacteric and nonclimacteric fruits, as well as in vegetative tissues. System‐2, by contrast, drives massive ethylene production during ripening, with levels increasing up to 300‐fold through autocatalytic regulation (Li *et al*., 2018; Pattyn *et al*., 2021; Huang *et al*., 2022). The transition from System‐1 to System‐2 occurs at the onset of fruit ripening, yet the underlying mechanism remains unclear (Chirinos *et al*., 2023).

The key enzymes of ethylene biosynthesis, 1‐aminocyclopropane‐1‐carboxyliase synthase (ACS) and 1‐aminocyclopropane‐1‐carboxylic acid oxidase (ACO), are encoded by multigene families, and their role in fruit growth and ripening has been widely studied. In tomato, *SlACS1* and *SlACS6*, involved in System‐1, are negatively regulated by ethylene, whereas *SlACS2* and *SlACS4* play a major role in System‐2 during fruit ripening and are upregulated through positive feedback by ethylene (Barry *et al*., 2000). *SlACO1* and *SlACO4* are expressed at low levels during fruit growth but are induced as ethylene levels rise at the onset of climacteric ripening (Liu *et al*., 2015).

There is a great diversity of tomato spontaneous ripening‐related mutants, including *ripening‐inhibitor* (*rin*), *nonripening* (*nor*), or *colorless nonripening* (*Cnr*), which affect the initiation of ripening, as well as *Never‐ripe* (*Nr*) and *Green‐ripe* (*Gr*), which are impaired in ethylene perception and/or signaling (Barry *et al*., 2005; Brumos, 2021). These mutants have enabled the identification of numerous transcription factors and genes involved in regulating ethylene biosynthesis, perception, and signaling pathways. Among them, three have emerged as key master regulators: *RIN*, a SEPALLATA MADS‐box transcription factor, *CNR*, a SQUAMOSA promoter binding protein, and *NOR*, an NAC transcription factor (Giovannoni *et al*., 2017; Li *et al*., 2018; Liu *et al*., 2024) (Fig. 4). Ethylene perception occurs through membrane‐bound ethylene receptors (ETRs), which act as negative regulators. In the absence of ethylene, active ETRs repress the hormone's signaling pathway, preventing the activation of downstream responses (Fig. 4). However, when ethylene binds to ETRs, the Constitutive Triple Response protein is inactivated. This leads to the activation of Ethylene‐Insensitive 2 (EIN2), which translocates to the nucleus and stabilizes Ethylene‐Insensitive 3 (EIN3) and EIN3‐like proteins (EILs). EIN3 and EILs upregulate the expression of the Ethylene Response factor (ERF) gene family, whose members in turn activate different ripening‐related genes controlling aroma, firmness, color, and other quality attributes (Liu *et al*., 2015). Interestingly, in melon, several mapping populations, including recombinant inbred lines and introgression lines (ILs), have been developed by crossing climacteric with nonclimacteric varieties (Pujol & Garcia‐Mas, 2023), allowing the map‐based cloning of some ripening‐related QTLs. To date, two transcription factors, *CmNAC‐NOR* and *CmERF024*, have been functionally validated by CRISPR/*Cas9* gene editing (Ríos *et al*., 2017; Liu *et al*., 2022; Santo Domingo *et al*., 2024). The *CmNAC‐NOR* gene, homologous to tomato *NOR*, produces comparable phenotypes in both species when disrupted, confirming its role as a master regulator of ripening both in tomato and melon. By contrast, no tomato *ERF* has been found to perform an analogous function to *CmERF024*, which is associated with chromatin accessibility and gene expression dynamics throughout fruit ripening (Santo Domingo *et al*., 2024). The recent development of gene‐edited melon mutants targeting ripening‐related genes, such as *CmROS1*, *CmCTR1‐like*, *CmNAC‐NOR*, and *CmERF024* (Giordano *et al*., 2022; Liu *et al*., 2022; Santo Domingo *et al*., 2024), provides valuable plant materials for investigating ethylene biosynthesis and its regulation in climacteric and nonclimacteric fruits, and warrants in‐depth study to clarify the link between respiration and ethylene at ripening onset. These findings underscore how ripening control extends beyond classical hormonal regulation to involve higher‐order chromatin remodeling and epigenetic modulation. Future research should integrate transcriptomic and epigenomic approaches to decipher how chromatin accessibility, histone modifications, and DNA methylation shape the transcriptional reprogramming that initiates and sustains fruit ripening.

## VIII. Ethylene and respiration during climacteric ripening: a chicken‐egg situation?

Climacteric ripening is defined by the synchronization of the respiratory burst with the ethylene peak during the fruit ripening process (Paul *et al*., 2012; Giovannoni *et al*., 2017). Table 1 compiles studies across species, highlighting not only which process rises first but also the factors underlying long‐standing controversies. Most reports describe a concurrent increase in both processes, followed by those locating the climacteric peak of respiration as an event before the increase in ethylene synthesis (Table 1), consistent with earlier generalizations (Paul *et al*., 2012; Giovannoni *et al*., 2017). However, variable results within the same species suggest that synchronization between respiration and the autocatalytic ethylene production depends on different factors, including genotype and environmental conditions. An important subject of debate is whether on‐ and off‐vine ripening alters respiratory behavior. Some authors proposed that climacteric respiration might be an artifact of harvest, rather than a key feature of ripening, in species such as melon, tomato and passion fruit (Saltveit Jr., 1993; Shellie & Saltveit, 1993; Shiomi *et al*., 1996). Yet other studies detected clear respiration bursts both on‐ and off‐vine in the same species (Sawamura *et al*., 1978; Andrews, 1995; Hadfield *et al*., 1995; Bower *et al*., 2002) and in additional species (Hulme *et al*., 1963; Nordey *et al*., 2016), indicating that observed variation likely reflects cultivar‐specific rather than methodological differences. Regarding ethylene, results are equally inconsistent; some studies report higher levels in attached fruits (Sfakiotakis & Dilley, 1973; Hadfield *et al*., 1995; Rogiers & Knowles, 1999; Bower *et al*., 2002), whereas others find the opposite (Saltveit Jr., 1993; Shellie & Saltveit, 1993; Ayub *et al*., 1996; Shiomi *et al*., 1996). Furthermore, detached fruits from several species have a faster ripening (Bower *et al*., 2002 & references therein), avocado being an extreme case as it is unable to ripen unless harvested from the tree (Hunter *et al*., 2024). Given these contrasting findings and the limited number of direct comparisons (Table 1), future research should systematically examine on‐ vs off‐vine ripening across diverse species to clarify how respiration and ethylene synthesis are coordinated.

**Table 1:** Compilation of studies investigating the patterns of respiration and ethylene production during climacteric ripening in various fleshy fruit species.

Species	Family	Ripening	Ethylene	Ethylene detection	Respiration	First process	References
Avocado (*Persea americana*)	Lauraceae	Off vine	External	Other	CO_2_	Both	Biale *et al*. (1954)
				Gas chromatography	CO_2_	Ethylene	Adato & Gazit (1977)
							Eaks (1983)
							Tucker & Laties (1984)
						Respiration	Awad & Young (1979)
			Internal	Gas chromatography	CO_2_	Ethylene	Burg & Burg (1962b)
			Both	Gas chromatography	CO_2_	Respiration	Kosiyachinda & Young (1975)
Banana (*Musa* spp.)	Musaceae	Off vine	External	Other	CO_2_	Both	Nelson (1939)
							Biale *et al*. (1954)
				Gas chromatography	O_2_	Both	McMurchie *et al*. (1972)
					CO_2_ and O_2_	Both	Liu *et al*. (2004)
			Both	Gas chromatography	CO_2_	Respiration	Burg & Burg (1962a)
Apple (*Malus domestica*)	Rosaceae	Off vine	External	Other	CO_2_	Respiration	Nelson (1940)
						Both	Sams & Conway (1984)
			Both	Gas chromatography	CO_2_	Both	Song & Bangerth (1996)
							Rudell *et al*. (2000)
European pear (*Pyrus communis*)	Rosaceae	Off vine	External	Other	CO_2_ and O_2_	Respiration	Hansen (1942)
				Gas chromatography	CO_2_	Both	Brandes & Zude‐Sasse (2019)
			Internal	Gas chromatography	CO_2_	Ethylene	Wang & Mellenthin (1972)
Papaya (*Carica papaya*)	Caricaceae	Off vine	External	Gas chromatography	CO_2_	Ethylene	Fabi *et al*. (2007)
						Both	Resende *et al*. (2012)
Tomato (*Solanum lycopersicum*)	Solanaceae	Both	External	Other	CO_2_	Ethylene	Spencer (1956)
				Gas chromatography	O_2_	Ethylene	Xu *et al*. (2012)
					CO_2_	Both	Gamrasni *et al*. (2020)
			Both	Gas chromatography	CO_2_	Respiration	Herner & Sink (1973)
			Internal	Gas chromatography	CO_2_	Ethylene	Sawamura *et al*. (1978)
Goldenberry (*Physalis peruviana*)	Solanaceae	Off vine	External	Gas chromatography	CO_2_	Ethylene	Trinchero *et al*. (1999)
Kiwifruit (Actinidia deliciosa)	Actinidiaceae	Off vine	External	Gas chromatography	CO_2_	Both	Antunes *et al*. (2000)
							Antunes (2007)
Pawpaw (*Asimina triloba*)	Annonaceae	Off vine	External	Gas chromatography	CO_2_	Both	Archbold & Pomper (2003)
Cherimoya (*Annona cherimola*)	Annonaceae	Off vine	External	Other	CO_2_	Respiration	Biale *et al*. (1954)
				Gas chromatography	CO_2_ and O_2_	Both	Solomos & Laties (1976)
					CO_2_	Respiration	Brown *et al*. (1988)
							Martinez *et al*. (1993)
			Both	Gas chromatography	CO2	Respiration	Kosiyachinda & Young (1975)
Custard apple (*Annona reticulata*)	Annonaceae	Off vine	External	Gas chromatography	CO_2_	Both	Broughton & Guat (1979)
						Respiration	Brown *et al*. (1988)
Sugar apple (*Annona squamosa*)	Annonaceae	Off vine	External	Gas chromatography	CO_2_	Ethylene	Brown *et al*. (1988)
Soursop (*Annona muricata*)	Annonaceae	Off vine	External	Gas chromatography	CO2	Respiration	Paull (1982)
					CO_2_ and O_2_	Respiration	Bruinsma & Paull (1984)
Melon (*Cucumis melo*)	Cucurbitaceae	Off vine	Internal	Gas chromatography	CO_2_ and O_2_	Both	Lyons *et al*. (1962)
			External	Gas chromatography	CO_2_	Both	Kitamura *et al*. (1975)
							Mallick *et al*. (1984)
		On vine	External	Gas chromatography	O_2_	Ethylene	Bower *et al*. (2002)
Feijoa (*Acca sellowiana*)	Myrtaceae	Off vine	External	Other	CO_2_	Respiration	Biale *et al*. (1954)
Mango (*Magnifera indica*)	Anacardiaceae	Off vine	External	Other	CO_2_	Respiration	Biale *et al*. (1954)
			Both	Gas chromatography	CO_2_	Both	Burg & Burg (1962a)
		Both	External	Gas chromatography	CO_2_ and O_2_	Ethylene / Both	Nordey *et al*. (2016)
Passion Fruit (*Passiflora edulis*)	Passifloraceae	Both	External	Gas chromatography	CO_2_	Both	Shiomi *et al*. (1996)
Blueberry (*Vaccinium* spp.)	Ericaceae	On vine	External	Gas chromatography	CO_2_	Both	Wang *et al*. (2022)

Eighteen species from a broad range of botanical families are represented. For each study, information is provided on ripening conditions (on vine or off vine), whether internal or external ethylene was measured, method used for ethylene detection (gas chromatography or other), type of respiratory measurement (CO_2_ production and/or O_2_ consumption), and the first physiological process detected to increase during the ripening process (ethylene, respiration, or both). References to the original studies are included in the last column.

Differences in analytical approaches to determine internal or external ethylene have also contributed to discrepancies. Earlier studies relied on manometric methods, while the introduction of gas chromatography enabled more accurate detection of trace ethylene levels (Bleecker & Kende, 2000). However, results still vary among species regarding which process initiates first (Table 1). In melon, most studies agree that ethylene surge either precedes or coincides with the respiratory burst, regardless of whether internal or external ethylene is measured (Table 1). Later studies suggested that different ripening processes (i.e. Chl degradation, flesh softening) exhibit different sensitivity thresholds to ethylene (Pech *et al*., 2008), and that sensitivity itself also changes during ripening (Flores *et al*., 2008), similar to findings in banana (Inaba & Nakamura, 1988) and apple (Johnston *et al*., 2009). The development of highly sensitive *in planta* ethylene determinations for melon, capable of detecting concentrations in the ppb range (Pereira *et al*., 2017), offers a promising tool for disentangling the timing and specificity of ethylene‐triggered processes.

Finally, the way respiration has been assessed may have also contributed to the conflicting results regarding its synchronization with ethylene. Respiration has historically been measured as CO_2_ production, whereas few studies have measured O_2_ consumption (Table 1). CO_2_‐based assays may present technical and biological artifacts derived from fruit transpiration or CO_2_ refixation (see Section IV), as noted by Andrews (1995). When O_2_ consumption has been recorded, alone or alongside CO_2_ release, most studies observed a rise in respiration preceding or accompanying ethylene accumulation (Table 1). While respiratory CO_2_ evolution largely reflects fluxes through the pentose phosphate pathway and the TCA cycle (Sweetlove *et al*., 2013), O_2_ uptake more directly reports oxidative phosphorylation and mitochondrial ATP production, although this relationship depends on the relative engagement of ARPs. Because a fraction of respiratory CO_2_ can be refixed, CO_2_‐based measurements may underestimate or delay the apparent onset of respiratory activity, whereas O_2_ consumption more accurately reflects the timing and intensity of mitochondrial metabolism; therefore, direct O_2_ assays, together or not with CO_2_‐based measurements, are recommended for future studies seeking to resolve respiration‐ethylene dynamics. Respiration supplies the carbon skeletons, reductants, and ATP required for the synthesis of nucleic acids, proteins, pigments, and flavor compounds, all in high demand during fruit ripening (Tucker, 1993). Moreover, ATP produced by oxidative phosphorylation supports the Yang cycle, which recycles methionine and sustains high ethylene production during climacteric ripening (Fig. 4). Thus, an increase in COX‐dependent respiration would be expected at the climacteric peak due to its ATP‐coupled nature. Yet, evidence suggested the engagement of non‐phosphorylating AOX and UCP pathways during this stage (Xu *et al*., 2012; Colombié *et al*., 2017). Because O_2_ consumption through ARPs yields little or no ATP (Fig. 3), their precise role in climacteric respiration remains unclear and speculative (Perotti *et al*., 2014; Colombié *et al*., 2017; Hewitt & Dhingra, 2020). Recent *in vivo* analyses have begun to address this long‐standing question. Our recent work using O_2_‐isotope discrimination in tomato has demonstrated that AOX activity peaks precisely at the breaker stage, directly supporting ethylene synthesis by maintaining redox balance and precursor supply (Iglesias‐Sanchez *et al*., 2025). These findings provide a physiological framework for the functional connection between AOX respiration and the onset of the climacteric stage, setting the stage for further dissection of ethylene‐mitochondrial crosstalk.

## IX. The relationships between climacteric and alternative respiration

Most studies on ARPs in fruits have focused on AOX (Hewitt & Dhingra, 2020), with little attention given to NDIIs and UCPs (Fig. 3). Early work suggested that NDIIs might oxidize cytosolic NADPH produced by NADP‐ME during tomato fruit ripening (Goodenough *et al*., 1985), but functional analyses of NDIIs in fruits are still lacking. For UCPs and AOXs, conflicting changes in transcript and protein levels have been reported in apple, tomato, mango, and papaya during ripening (Almeida *et al*., 1999; Considine *et al*., 2001; Holtzapffel *et al*., 2002; Xu *et al*., 2012; Oliveira *et al*., 2015). Such discrepancies likely reflect differences between on‐ and off‐vine ripening (Almeida *et al*., 1999; Holtzapffel *et al*., 2002; Oliveira *et al*., 2015; Colombié *et al*., 2017) since metabolite patterns also differ markedly between both conditions (Osorio *et al*., 2020), potentially involving regulators of ARPs expression (i.e. citrate for AOX). Although ARPs expression typically peaks between the climacteric and the postclimacteric stages, *in vivo* fluxes through AOX and COX pathways rarely parallel transcript or protein abundance (Del‐Saz *et al*., 2018).

Early investigations by Solomos, Laties, and collaborators linked the AOX pathway with climacteric rise in respiration and ethylene production (Laties, 1982). Although inhibitor‐based methods used at that time are now considered inadequate for quantifying AOX and COX activities (Del‐Saz *et al*., 2018), they revealed that ethylene‐induced respiratory enhancement requires a functional AOX. Measurements of AOX activity based on inhibition (e.g. using salicylhydroxamic acid, SHAM) assays tend to underestimate AOX activity; consequently, it is now widely accepted that true *in vivo* AOX and COX activities can only be quantified using inhibitor‐free measurements with the oxygen isotope discrimination technique, whereas inhibitor‐derived rates can only be used to determine the capacities (maximum electron fluxes) of the two pathways (Del‐Saz *et al*., 2018). Later studies suggested that the climacteric rise in respiration may be associated with AOX activation by increased pyruvate accumulation and reducing power, both known AOX activators, resulting from enhanced glycolysis (reviewed in Hewitt & Dhingra, 2020; Fig. 4). Our recent work, combining O_2_ isotope discrimination with transcript and metabolomic analyses, provides direct *in vivo* evidence for this mechanism (Iglesias‐Sanchez *et al*., 2025). At the climacteric‐breaker stage, the increased supply of TCA cycle intermediates (pyruvate, 2‐oxoglutarate and succinate) allosterically triggered AOX *in vivo* activity, allowing enhanced NAD(P)H reoxidation to ensure carbon supply for triggering ethylene biosynthesis. AOX deficiency in newly generated AOX1a mutants restricted respiratory metabolism and specifically reduced aspartate and methionine pools, showing that AOX ensures the carbon and redox supply required for ethylene biosynthesis. These findings establish a synergistic relationship between AOX activity and ethylene production at the onset of ripening, where enhanced AOX flux sustains the transition to System‐2 ethylene synthesis. At later stages, AOX transcripts remain high but *in vivo* activity declines, likely reflecting posttranslational control and ROS‐dependent mitochondrial retrograde signaling rather than direct metabolic engagement. This pattern suggests that while AOX contributes to initiating ripening, other alternative components may become more relevant in maintaining redox balance during late ripening and senescence.

Beyond their direct metabolic roles, ARPs also influence mitochondrial retrograde regulation (MRR) of nuclear gene expression (Fig. 4) by modulating the levels of signaling molecules, with mitochondrial ROS being the primary trigger (Selinski *et al*., 2024). Among different NAC transcription factors, ANAC017 functions as a master regulator of MRR in Arabidopsis, inducing *AOX1a* and related genes (Selinski *et al*., 2024). Because AOX modulates ROS and RNS levels (Gupta *et al*., 2018), it acts as both target and regulator of retrograde signals (Fig. 4). ROS typically peak at the onset of ripening and during postripening senescence (Muñoz & Munné‐Bosch, 2018), suggesting ARPs could autoregulate their own expression via retrograde feedback.

Several NAC transcription factors controlling fruit ripening have been reported in tomato (Chen *et al*., 2023), yet none has been identified as a functional ortholog of ANAC017. Nevertheless, membrane‐tethered NAC proteins structurally related to ANAC017 occur in tomato, including SlSRN1 (Solyc12g056790) and Solyc04g072220, which possess N‐terminal NAC domains and C‐terminal transmembrane regions and cluster phylogenetically with ANAC017‐like proteins (Liu *et al*., 2014). These features make them attractive candidates for MRR‐type regulators in Solanaceae. Future studies should test whether such NACs mediate AOX regulation and MRR in fruit tissues. In Arabidopsis, ANAC017 also coordinates mitochondrial function with ethylene and auxin signaling via the MKK9–MPK3/6 cascade, integrating stress responses with growth (He *et al*., 2022). Among its downstream targets is a 2‐oxoglutarate/Fe(II)‐dependent oxygenase homologous to tomato E8, an ethylene‐induced oxygenase involved in α‐tomatine detoxification. Suppression of E8 increases ethylene production and alters redox balance, suggesting that E8‐like oxygenases connect ethylene‐responsive detoxification and redox regulation during ripening (D. Li *et al*., 2025), possibly interfacing indirectly with MRR through redox control.

In Arabidopsis seedlings, ANAC017 and AOX1a contribute to the ethylene‐induced triple response phenotype (Merendino *et al*., 2020). Nitric oxide (NO), generated under hypoxia, may link AOX and ethylene, since NO inhibits ethylene‐induced fruit ripening via several mechanisms including transcriptional and posttranslational regulation of ethylene biosynthetic enzymes (Manjunatha *et al*., 2012; Fig. 4). Unlike COX, AOX is resistant to NO inhibition and is instead transcriptionally induced by it (Gupta *et al*., 2018), reinforcing its role in mitochondrial‐ethylene crosstalk (Fig. 4). Cyanide, a by‐product of ethylene synthesis, may also contribute to retrograde signaling when produced at low, sustained levels despite detoxification systems (Xu *et al*., 2023). Furthermore, growing evidence supports a role for ethylene in regulating RNS and ROS levels through AOX activity during hypoxia (Zafari *et al*., 2022), a condition occurring in specific internal fruit tissues (X. Li *et al*., 2025). Collectively, these observations reveal a complex feedback network in which mitochondrial function, redox homeostasis, and ethylene signaling are intimately interconnected during ripening.

## X. Respiration and secondary metabolism in fruits

**Fig. 5:**
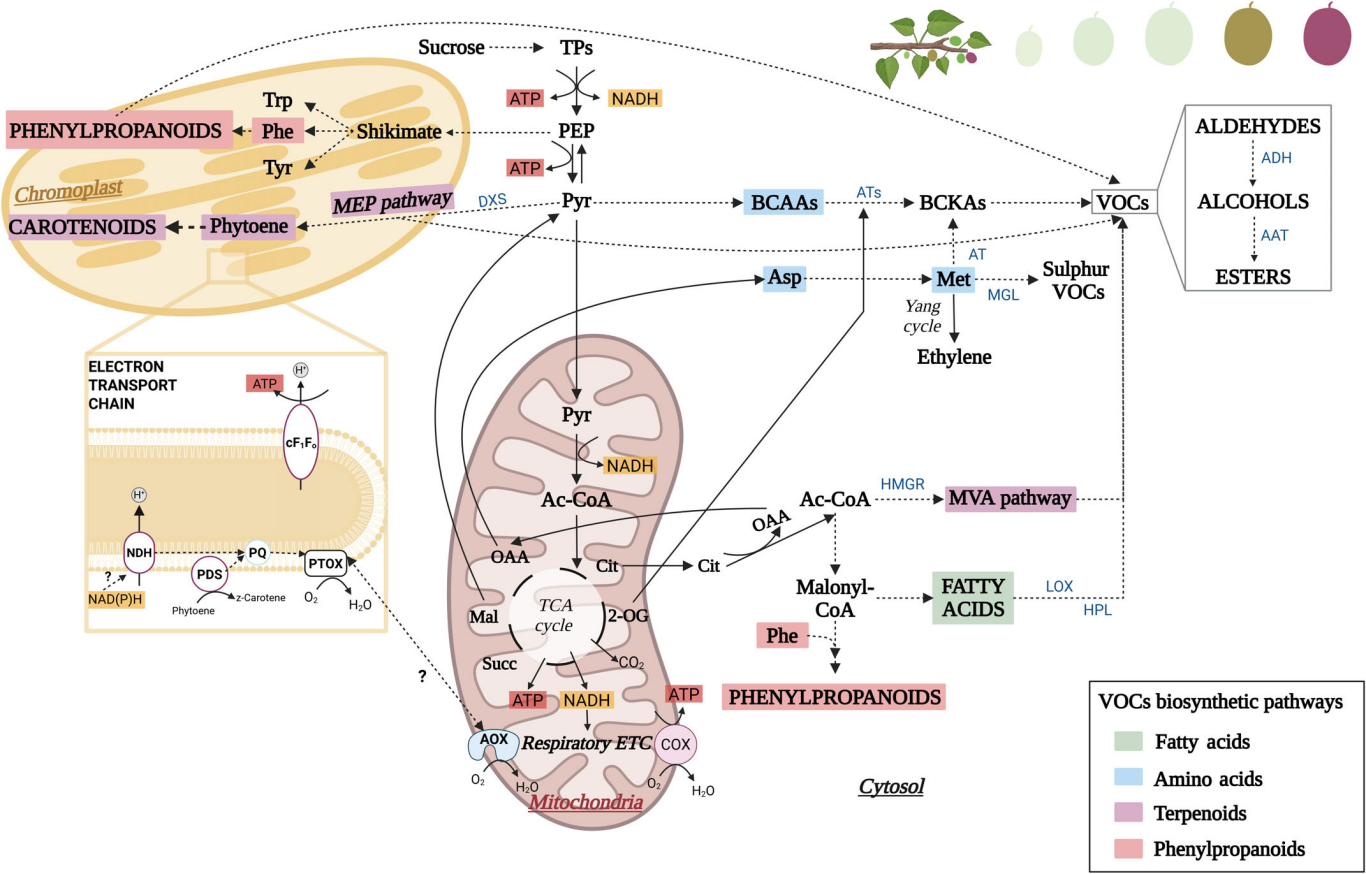
Schematic overview of the interactions between respiratory and secondary metabolism during fruit ripening. Respiratory metabolism supplies both energy and carbon precursors for the synthesis of secondary metabolites, including phenylpropanoids, carotenoids, and volatile organic compounds (VOCs), via the shikimate, methylerythritol phosphate (MEP), mevalonate (MVA), and fatty acid β‐oxidation pathways. The principal respiratory precursors feeding these pathways are glycolytic intermediates (TPs, triose phosphates; PEP, phosphoenolpyruvate; Pyr, pyruvate), acetyl‐CoA (Ac‐CoA), and several TCA cycle intermediates (Cit, citrate; 2‐OG, 2‐oxoglutarate; OAA, oxalacetate). These interfaces between respiratory and secondary metabolism are detailed in the main text; the key enzymes involved are highlighted in blue in the figure: 1‐deoxy‐D‐xylulose 5‐phosphate synthase (DXS), 3‐hydroxy‐3‐methylglutaryl‐CoA (HMGR), aminotransferases (ATs), lipoxygenase (LOX), hydroperoxide lyase (HPL), alcohol dehydrogenase (ADH), alcohol acyl transferase (AAT). Additional abbreviations: plastidial NADH dehydrogenase complex (NDH); phytoene desaturase (PDS); plastoquinone (PQ); plastid terminal oxidase (PTOX); chloroplast ATP synthase (cF_1_F_o_); malate (Mal); succinate (Succ); tryptophan (Trp); tyrosine (Tyr); phenylalanine (Phe); aspartate (Asp); methionine (Met); branched‐chain amino acids (BCAAs); branched‐chain α‐ketoacids (BCKAs). Background colors denote metabolites or pathways involved in the four different main VOC biosynthetic routes: green, fatty‐acid‐derived VOCs; blue, amino‐acid‐derived VOCs; pink, terpenoid VOCs; red, phenylpropanoid‐derived VOCs. This figure was created in BioRender, Pujol M. (2026) (https://BioRender.com/dwdd7d9).

Fleshy fruits synthesize numerous health‐related and economically relevant metabolites, including primary metabolism compounds (i.e. ascorbate or vitamin C), and a broad range of secondary metabolites, including carotenoids (i.e. β‐carotene or pro‐vitamin A), volatiles, and phenylpropanoids (Fig. 5; Fenech *et al*., 2019; Zhu *et al*., 2022; Rodriguez‐Concepcion *et al*., 2018). As a general link to respiratory metabolism, glycolytic intermediates provide the carbon skeletons for phenylpropanoid, carotenoid, and volatile biosynthesis via the shikimate, methylerythritol phosphate (MEP), mevalonate (MVA), and fatty acid β‐oxidation pathways (Fig. 5). However, the specific contributions of different respiratory pathways to the accumulation of these compounds remain only partially resolved (Pott *et al*., 2019; Zhu *et al*., 2022; McDonald, 2023).

Hundreds of volatile organic compounds (VOCs) including aldehydes, alcohols, esters, terpenoids, and phenylpropanoids accumulate during fruit ripening, all derived from primary metabolism (Gonda *et al*., 2016; Pott *et al*., 2019; Fig. 5). Hence, precursor availability and its regulation by respiration are key determinants of fruit aroma. The main VOCs biosynthetic routes are the fatty acid, the amino acid, and the terpenoid pathways (Fig. 5). Since the 1960s, the climacteric respiratory burst has been linked to an increase in lipids and free fatty acids, which subsequently decline as they are metabolized into VOCs. Those studies set the basis for fruit storage management under controlled atmosphere (Song & Bangerth, 2003). Fatty acid‐derived VOCs depend on the activity of lipoxygenases (LOX) and hydroperoxide lyases (HPL). LOX genes form large families: 23 putative members in apple (Vogt *et al*., 2013), 14 in tomato (Upadhyay & Mattoo, 2018), and 18 in melon (Zhang *et al*., 2014), but only a subset contributes to VOCs production. Thus, LOXs represent prime targets for metabolic engineering, although more studies are needed to determine whether their transcript abundance limits VOCs synthesis. Notably, increased fatty acid precursors such as linolenic acid caused an increase in some aldehydes, alcohols, and esters in kiwi, melon, tomato, pear, or apple (Zhang *et al*., 2009; Ties & Barringer, 2012; Tang *et al*., 2015; Contreras *et al*., 2016). However, the strongest aroma modification in pear occurred upon increasing secondary intermediates that are direct precursors of esters (Qin *et al*., 2014).

As another important group, amino acid‐derived VOCs are produced from methionine, phenylalanine, and the branched‐chain amino acids (BCAA) leucine, valine, and isoleucine (Fig. 5). Even though there is not much information about the first steps in this biosynthetic pathway, in melon three aminotransferases (*CmBCAT1*, *CmArAT1*, and *CmMetAT*) have been identified that convert amino acids into α‐ketoacids, and their expression levels correlate with the abundance of amino acid‐derived VOCs (Gonda *et al*., 2010, 2026; Li *et al*., 2016). However, in tomato or grape, *BCAT1* overexpression did not affect volatile levels (Kochevenko *et al*., 2012), and phenylalanine content was not correlated with phenylalanine‐derived VOCs (Dal Cin *et al*., 2011). While BCAA catabolism contributes to respiration during fruit development, it appears negligible in mature fruits (Kochevenko *et al*., 2012). Given the central role of amino acids in overall metabolism, altering their abundance is unlikely to selectively change fruit aroma, making metabolic engineering of these pathways challenging. Once the biosynthetic pathways and the regulatory network are defined, more precise engineering may become feasible. The phenylpropanoid pathway produces both aromatic VOCs and nonvolatile derivatives such as flavonoids. Phenylalanine is deaminated by phenylalanine ammonia lyase to cinnamic acid, leading to the synthesis of phenylpropanoids, phenylpropenes, phenylpropanes, and volatile benzenoids (Gonda *et al*., 2018). In melon, genes such as *CmCNL* and *CmBAMT* have been identified within this pathway (Mayobre *et al*., 2024). A parallel methionine degradation pathway produces sulfur volatiles via methionine‐γ‐lyase enzyme, linked to isoleucine metabolism (Maoz *et al*., 2022; Fig. 5).

Terpenoids are the most structurally diverse group of volatiles, contributing to flower and fruit aroma, as well as to defense signaling (Sharma *et al*., 2017; Mayobre *et al*., 2024). They are synthesized through the MVA pathway in the cytosol from acetyl CoA and the MEP pathway in plastids from pyruvate and glyceraldehyde‐3‐phosphate (GAP) (Fig. 5). Both pathways produce isoprenoid units that, through prenyltransferases, will produce the precursors of monoterpenes, sesquiterpenes, or apocarotenoids. The key MVA pathway enzyme 3‐hydroxy‐3‐methylglutaryl CoA (HMGR) catalyzes MVA formation and is a proven engineering target: HMGR overexpression increased phytosterol content in tomato (Enfissi *et al*., 2005). In the MEP pathway, 1‐deoxy‐D‐xylulose 5‐phosphate synthase (DXS) catalyzes the condensation of pyruvate and GAP, followed by the isomerization to MEP by DXP reductoisomerase (DXR). Coordinated regulation of DXS and phytoene synthase (PSY1) links terpenoid and carotenoid biosynthesis; their co‐expression correlates with elevated carotenoid levels in tomato, pepper, orange, or orange‐fleshed melons (Saladié *et al*., 2014; Pott *et al*., 2019). Overexpressing DXS in grape similarly enhanced monoterpene content (Dalla Costa *et al*., 2018). Although ethylene‐mediated transcriptional cascades regulating VOC production are well established, the direct contribution of respiration to volatile biosynthesis, particularly via precursor supply, remains to be clarified.

Respiratory metabolism is also critical for producing carotenoids in plastids of nonphotosynthetic tissues. During ripening of many fleshy fruits, chloroplasts differentiate into chromoplasts, losing thylakoid structures, and remodeling the internal membrane system, in parallel with a massive accumulation of carotenoids in plastoglobules (Sadali *et al*., 2019). As the Calvin cycle becomes inactive, the MEP pathway relies on pyruvate and GAP from glycolysis (Fig. 5; Bouvier & Camara, 2007). Hexoses derived from plastid starch breakdown and cytosolic imports likely support the high carbon demand for carotenoid synthesis in tomato fruit chromoplasts (Bouvier & Camara, 2007). Respiratory pathways in the cytosol and mitochondria may also supply ATP for ensuring the plastid MEP pathway, as shown in poplar leaves and thermogenic plants (Loreto *et al*., 2007; Terry *et al*., 2016). In tomato, plastid terminal oxidase (PTOX)‐dependent chromorespiration produces ATP within fruit chromoplasts (Renato *et al*., 2014; Fig. 5). While this ATP production can be relevant at later developmental stages (Renato *et al*., 2014), the *in vivo* contribution of mito‐ vs chromo‐respiration during fruit ripening has only recently begun to be unresolved. Our recent work demonstrated that mitochondrial AOX activity peaks at the breaker stage, preceding maximal carotenoid accumulation (Iglesias‐Sanchez *et al*., 2025). This climacteric burst of nonphosphorylating AOX respiration sustains high glycolytic and TCA cycle fluxes, ensuring the supply of key metabolic precursors such as pyruvate for carotenoid biosynthesis, and aspartate and methionine for ethylene production. As ripening progresses and AOX activity declines, plastidial oxidases such as PTOX appear to assume a complementary role, providing ATP and maintaining redox balance required for the phytoene‐to‐lycopene desaturation steps (Renato *et al*., 2014). The plastidial NADH dehydrogenase (NDH) complex is also essential for sustaining carotenoid synthesis in tomato (Nashilevitz *et al*., 2010; Fig. 5). Together, these results reveal a coordinated handover between mitochondrial and plastidial alternative respiratory activities that collectively support the metabolic and energetic demands of chromoplast differentiation during fruit ripening.

Finally, research into the interconnection of fruit respiration and flavonoid production is the subject of scarce direct research. Flavonoids derive primarily from shikimate and acetate pathways (Saito *et al*., 2013; Perez de Souza *et al*., 2020) with acetyl CoA tightly connected to the TCA cycle through redox‐dependent regulation of ATP‐citrate lyase (Daloso *et al*., 2015). That said, integrating insights from primary and specialized metabolism across fruit ripening (Carrari & Fernie, 2006) reveals that the peak of flavonoid production coincides with the climacteric respiration burst. This temporal overlap suggests that flavonoid production is greatly influenced by precursor supply. This hypothesis is strongly supported by the finding that the overexpression of two transcription factors in tomato increased both anthocyanin content (Butelli *et al*., 2008) and expression of genes associated with the primary metabolic pathways which supplied their precursors (Tohge *et al*., 2015; Zhang *et al*., 2015). However, this is likely only one of many links between respiration and flavonoid production. Whilst genome‐wide association studies have identified a plethora of genes associated with flavonoid production (Ding *et al*., 2023; Chen *et al*., 2024; Lai *et al*., 2024; C. Li *et al*., 2025), their potential for exploring respiration–flavonoid coordination remains largely untapped (Bulut *et al*., 2023). That said, once such datasets exist it will be facile to dissect the importance of respiration regarding the accumulation of flavonoids.

## XI. Conclusions and future perspectives

Although fruit respiration has been studied for nearly a century, the role and regulation of ARPs during fruit growth and their impact on carbon metabolism (Figs 1, 2), including photosynthesis, remain largely overlooked. Interactions between fruit photosynthesis and respiration (Fig. 2), particularly under stress conditions, warrant greater attention in the context of climate change. While reducing respiratory carbon loss is often viewed as a strategy to enhance plant growth, the distinctive features of fruit metabolism, such as high CO_2_ refixation capacity and possible involvement of ARPs in stress tolerance, suggest that stimulating respiration could instead promote fruit growth and potentially improve fruit quality and stress resilience.

Research on fruit respiration has largely focused on the climacteric phase, where ARPs are recognized as key players in ethylene‐triggered ripening (Fig. 4). Metabolic models predicted a significant contribution of AOX and UCP pathways (Colombié *et al*., 2017), yet experimental *in vivo* evidence for their regulation and function remained scarce until recently. Our recent work provided the first *in vivo* quantification of AOX and COX engagement during tomato ripening, showing that AOX activity peaks at the breaker stage, when respiration and ethylene synthesis are tightly coupled (Iglesias‐Sanchez *et al*., 2025). At this stage, enhanced AOX flux ensures the carbon and redox supply required for aspartate‐ and methionine‐dependent ethylene biosynthesis, establishing a synergistic relationship between AOX activity and ethylene production that sustains the transition to System‐2 ethylene synthesis. At later stages, AOX activity declines despite sustained transcript levels, suggesting a switch toward posttranslational and ROS‐mediated mitochondrial retrograde signaling. In parallel, plastidial PTOX activity becomes increasingly important for maintaining redox balance and carotenoid desaturation during advanced ripening. Together, these findings redefine the interplay between mitochondrial and plastidial respiration as a dynamic continuum rather than an antagonistic trade‐off between pathways. Compared to AOXs, NDIIs and UCPs remain understudied despite their likely importance. To unravel ARP coordination, genetic studies should target multiple gene families, particularly those with fruit‐specific expression patterns.

Collectively, physiological and modeling evidence indicate that carbon fluxes in fleshy fruits shift progressively from predominantly catabolic to partially gluconeogenic modes as ripening advances. Organic acids that accumulate during growth act initially as storage and anaplerotic sinks, but are later remobilized through PEPCK‐ and PPDK‐dependent pathways to sustain sugar and secondary‐metabolite synthesis once respiratory demand declines. This transition underscores the tight interplay between vacuolar compartmentation, redox and pH regulation, and mitochondrial activity, integrating respiratory and biosynthetic metabolism across developmental stages (Walker *et al*., 2021). As described in Section X, this metabolic flexibility directly supports the formation of carotenoids, phenylpropanoids, and volatiles, with respiratory and alternative pathways supplying both the carbon skeletons and the reducing power required for their biosynthesis. Understanding how these fluxes are coordinated with ARP activity will be key to linking primary and specialized metabolism during ripening.

A complex molecular crosstalk connects AOX respiration, MRR, and ethylene signaling (Fig. 4), likely involving feedback mechanisms that fine‐tune gene expression and contribute to ripening. This area demands deeper investigation, particularly to define how ROS, RNS, and redox‐sensitive NAC transcription factors mediate communication between mitochondria and the nucleus. Studies of respiratory metabolism in mutants with altered ethylene production, either reduced (e.g. *rin*, *cnr*, *nor*) or enhanced, will be essential to clarifying the hierarchy of events driving ripening. Notably, all AOXs in the Cucurbitaceae family are of the AOX2 type, with only a single gene copy reported in most species analyzed (Costa *et al*., 2014). *Cucumis melo* is unique among fleshy fruits for possessing both climacteric and nonclimacteric varieties but only one AOX2 isoform, making it an ideal model for dissecting AOX function across ripening types. Now that *in vivo* electron partitioning between AOX and COX can be quantified during ripening (Iglesias‐Sanchez *et al*., 2025), applying this approach to climacteric and nonclimacteric melon genotypes would enable a direct comparison of ARP engagement in both ripening types. Such analyses could reveal whether tight AOX‐ethylene coupling is a specific hallmark of climacteric behavior or a more general feature of fruit ripening.

Beyond genetic and molecular regulation, the spatial dimension of fruit respiration deserves greater focus. Distinct tissues and cell types display contrasting metabolic roles, as shown in tomato where the pericarp and locular tissues differ markedly in organic acid and sugar composition, reflecting divergent carbon fluxes (Lemaire‐Chamley *et al*., 2019). Comparable compartmentation occurs in cucumber, where inner fruit tissues exhibit stronger internal CO_2_ refixation than outer tissues, consistent with differences in photosynthetic and respiratory activity (Garrido *et al*., 2023). Recent isotopic‐labeling and metabolomic analyses in tomato further reveal that internal tissues such as the columella sustain higher glycolytic and TCA cycle‐associated fluxes under low O_2_, whereas outer tissues maintain a more balanced redox metabolism (X. Li *et al*., 2025). These results provide direct evidence that O_2_ gradients shape tissue‐specific respiratory activity within the fruit. Dedicated studies of tissue‐specific respiratory metabolism, including ARP regulation, will be essential to understand how these spatial patterns coordinate carbon use and energy efficiency during fruit development and postharvest life. Components of the ARPs, including AOX, NDIIs, and UCPs, are differentially activated under O_2_ limitation and reoxygenation to maintain redox balance, limit ROS and NO accumulation, and modulate O_2_‐sensing pathways (Jethva *et al*., 2023; Selinski *et al*., 2024). Their selective engagement across fruit tissues may represent a key mechanism of respiratory adaptation to heterogeneous oxygen microenvironments.

Despite remaining knowledge gaps, targeted manipulation of ARPs holds promise for developing ripening control and postharvest strategies that regulate respiration directly, offering an alternative or complement to the current ethylene‐centered approaches widely used in the industry. Future efforts should prioritize generating crop varieties with optimized AOX activity to support proper fruit development and ripening, while elucidating the functional interplay among AOX, COX, PTOX, and UCPs. Comparative studies of on‐ and off‐vine ripening across species could also yield valuable insights for improving postharvest technologies. Finally, clarifying how respiratory metabolism regulates secondary metabolite biosynthesis remains a major open question; elucidating these networks will be essential for future metabolic engineering approaches aimed at enhancing the accumulation of health‐promoting metabolites in fleshy fruits.

## Competing interests

None declared.

## Author contributions

AI‐S and SG‐C contributed equally to this work.

## Disclaimer

The New Phytologist Foundation remains neutral with regard to jurisdictional claims in maps and in any institutional affiliations.

## References

[nph70882-bib-0160] Adato I , Gazit S . 1977. Role of ethylene in avocado fruit development and ripening: II. Ethylene production and respiration by harvested fruits. Journal of Experimental Botany 28: 644–649.

[nph70882-bib-0001] Almeida AM , Jarmuszkiewicz W , Khomsi H , Arruda P , Vercesi AE , Sluse FE . 1999. Cyanide‐resistant, ATP‐synthesis‐sustained, and uncoupling‐protein‐sustained respiration during postharvest ripening of tomato fruit. Plant Physiology 119: 1323–1329. 10198091 10.1104/pp.119.4.1323 PMC32017

[nph70882-bib-0002] Amthor JS . 2023. ATP yield of plant respiration: potential, actual and unknown. Annals of Botany 132: 133–162. 37409716 10.1093/aob/mcad075 PMC10550282

[nph70882-bib-0003] Andrews J . 1995. The climacteric respiration rise in attached and detached tomato fruit. Postharvest Biology and Technology 6: 287–292.

[nph70882-bib-0161] Antunes MDC . 2007. The role of ethylene in kiwifruit ripening and senescence. Stewart Postharvest Review 3: 1–8.

[nph70882-bib-0162] Antunes MDC , Pateraki I , Kanellis AK , Sfakiotakis EM . 2000. Differential effects of low‐temperature inhibition on the propylene induced autocatalysis of ethylene production, respiration and ripening of ‘Hayward’ kiwifruit. The Journal of Horticultural Science and Biotechnology 75: 575–580.

[nph70882-bib-0004] Arabia A , Munné‐Bosch S , Muñoz P . 2024. Ascorbic acid as a master redox regulator of fruit ripening. Postharvest Biology and Technology 207: 112614.

[nph70882-bib-0163] Archbold DD , Pomper KW . 2003. Ripening pawpaw fruit exhibit respiratory and ethylene climacterics. Postharvest Biology and Technology 30: 99–103.

[nph70882-bib-0164] Awad M , Young RE . 1979. Postharvest variation in cellulase, polygalacturonase, and pectinmethylesterase in avocado (*Persea americana* Mill, cv. Fuerte) fruits in relation to respiration and ethylene production. Plant Physiology 64: 306–308. 16660954 10.1104/pp.64.2.306 PMC543076

[nph70882-bib-0005] Ayub R , Guis M , Ben Amor M , Gillot L , Roustan JP , Latché A , Bouzayen M , Pech JC . 1996. Expression of ACC oxidase antisense gene inhibits ripening of cantaloupe melon fruits. Nature Biotechnology 14: 862–866. 10.1038/nbt0796-862 9631011

[nph70882-bib-0006] Barreto P , Couñago RM , Arruda P . 2020. Mitochondrial uncoupling protein‐dependent signaling in plant bioenergetics and stress response. Mitochondrion 53: 109–120. 32439620 10.1016/j.mito.2020.05.001

[nph70882-bib-0007] Barreto P , Feitosa‐Araujo E , Fernie AR , Schwarzländer M . 2025. How to turbo charge respiration‐thermogenic metabolism in plants. Current Opinion in Plant Biology 85: 102730. 40311169 10.1016/j.pbi.2025.102730

[nph70882-bib-0008] Barry CS , Giovannoni JJ . 2007. Ethylene and fruit ripening. Journal of Plant Growth Regulation 26: 143–159.

[nph70882-bib-0009] Barry CS , Llop‐Tous MI , Grierson D . 2000. The regulation of 1‐aminocyclopropane‐1‐carboxylic acid synthase gene expression during the transition from system‐1 to system‐2 ethylene synthesis in tomato. Plant Physiology 123: 979–986. 10889246 10.1104/pp.123.3.979 PMC59060

[nph70882-bib-0010] Barry CS , McQuinn RP , Thompson AJ , Seymour GB , Grierson D , Giovannoni JJ . 2005. Ethylene insensitivity conferred by the Green‐ripe and never‐ripe 2 ripening mutants of tomato. Plant Physiology 138: 267–275. 15834010 10.1104/pp.104.057745 PMC1104181

[nph70882-bib-0011] Batista‐Silva W , Nascimento VL , Medeiros DB , Nunes‐Nesi A , Ribeiro DM , Zsögön A , Araújo WL . 2018. Modifications in organic acid profiles during fruit development and ripening: correlation or causation? Frontiers in Plant Science 9: 1689. 30524461 10.3389/fpls.2018.01689 PMC6256983

[nph70882-bib-0012] Beauvoit B , Belouah I , Bertin N , Cakpo CB , Colombié S , Dai Z , Gautier H , Génard M , Moing A , Roch L *et al*. 2018. Putting primary metabolism into perspective to obtain better fruits. Annals of Botany 122: 1–21. 29718072 10.1093/aob/mcy057 PMC6025238

[nph70882-bib-0165] Biale JB , Young RE , Olmstead AJ . 1954. Fruit respiration and ethylene production. Plant Physiology 29: 168. 16654632 10.1104/pp.29.2.168 PMC540484

[nph70882-bib-0013] Biale JB , Young RE . 1981. Respiration and ripening in fruits‐retrospect and prospect. In: Friend J , Rhodes MJC , eds. Recent advances in the biochemistry of fruits and vegetables. Orlando, FL, USA: Academic Press, 1–39.

[nph70882-bib-0014] Bleecker AB , Kende H . 2000. Ethylene: a gaseous signal molecule in plants. Annual Review of Cell and Developmental Biology 16: 1–18. 10.1146/annurev.cellbio.16.1.1 11031228

[nph70882-bib-0015] Bouvier F , Camara B . 2007. The role of plastids in ripening fruits. In: Wise RR , Hoober JK , eds. The structure and function of plastids. Advances in photosynthesis and respiration. Dordrecht, the Netherlands: Springer, 419–432.

[nph70882-bib-0016] Bower J , Holford P , Latché A , Pech JC . 2002. Culture conditions and detachment of the fruit influence the effect of ethylene on the climacteric respiration of melon. Postharvest Biology and Technology 26: 135–146.

[nph70882-bib-0166] Brandes N , Zude‐Sasse M . 2019. Respiratory patterns of European pear (*Pyrus communis L*.‘Conference’) throughout pre‐and post‐harvest fruit development. Heliyon 5: e01160. 30775567 10.1016/j.heliyon.2019.e01160 PMC6357215

[nph70882-bib-0167] Broughton WJ , Guat T . 1979. Storage conditions and ripening of the custard apple *Annona squamosa* L. Scientia Horticulturae 10: 73–82.

[nph70882-bib-0168] Brown BI , Wong LS , George AP , Nissen RJ . 1988. Comparative studies on the postharvest physiology of fruit from different species of *Annona* (custard apple). Journal of Horticultural science 63: 521–528.

[nph70882-bib-0169] Bruinsma J , Paull RE . 1984. Respiration during postharvest development of soursop fruit, *Annona muricata* L. Plant Physiology 76: 131–138. 16663783 10.1104/pp.76.1.131 PMC1064242

[nph70882-bib-0017] Brumos J . 2021. Gene regulation in climacteric fruit ripening. Current Opinion in Plant Biology 63: 102042. 33971378 10.1016/j.pbi.2021.102042

[nph70882-bib-0018] Bulut M , Alseekh S , Fernie AR . 2023. Natural variation of respiration‐related traits in plants. Plant Physiology 191: 2120–2132. 36546766 10.1093/plphys/kiac593 PMC10069898

[nph70882-bib-0019] Burbidge CA , Ford CM , Melino VJ , Wong DCJ , Jia Y , Jenkins CLD , Soole KL , Castellarin SD , Darriet P , Rienth M *et al*. 2021. Biosynthesis and cellular functions of tartaric acid in grapevines. Frontiers in Plant Science 12: 643024. 33747023 10.3389/fpls.2021.643024 PMC7970118

[nph70882-bib-0171] Burg SP , Burg EA . 1962a. Role of ethylene in fruit ripening. Plant Physiology 37: 179–189. 16655629 10.1104/pp.37.2.179 PMC549760

[nph70882-bib-0170] Burg SP , Burg EA . 1962b. Post‐harvest ripening of avocados. Nature 194: 398–399.

[nph70882-bib-0020] Butelli E , Titta L , Giorgio M , Mock HP , Matros A , Peterek S , Schijlen EG , Hall RD , Bovy AG , Luo J *et al*. 2008. Enrichment of tomato fruit with health‐promoting anthocyanins by expression of select transcription factors. Nature Biotechnology 26: 1301–1308. 10.1038/nbt.1506 18953354

[nph70882-bib-0021] Carrari F , Baxter C , Usadel B , Urbanczyk‐Wochniak E , Zanor MI , Nunes‐Nesi A , Nikiforova V , Centero D , Ratzka A , Pauly M *et al*. 2006. Integrated analysis of metabolite and transcript levels reveals the metabolic shifts that underlie tomato fruit development and highlight regulatory aspects of metabolic network behavior. Plant Physiology 142: 1380–1396. 17071647 10.1104/pp.106.088534 PMC1676044

[nph70882-bib-0022] Carrari F , Fernie AR . 2006. Metabolic regulation underlying tomato fruit development. Journal of Experimental Botany 57: 1883–1897. 16449380 10.1093/jxb/erj020

[nph70882-bib-0023] Centeno DC , Osorio S , Nunes‐Nesi A , Bertolo ALF , Carneiro RT , Araújo WL , Steinhauser M , Michalska J , Rohrmann J , Geigenberger P *et al*. 2011. Malate plays a crucial role in starch metabolism, ripening, and soluble solid content of tomato fruit and affects postharvest softening. Plant Cell 23: 162–184. 21239646 10.1105/tpc.109.072231 PMC3051241

[nph70882-bib-0024] Chen J , Zhang Y , Wei J , Hu X , Yin H , Liu W , Li D , Tian W , Hao Y , He Z *et al*. 2024. Beyond pathways: Accelerated flavonoids candidate identification and novel exploration of enzymatic properties using combined mapping populations of wheat. Plant Biotechnology Journal 22: 2033–2050. 38408119 10.1111/pbi.14323 PMC11182594

[nph70882-bib-0025] Chen N , Shao Q , Lu Q , Li X , Gao Y , Xiao Q . 2023. Research progress on function of NAC transcription factors in tomato (*Solanum lycopersicum* L.). Euphytica 219: 22.

[nph70882-bib-0026] Chen T , Qin G , Tian S . 2020. Regulatory network of fruit ripening: current understanding and future challenges. New Phytologist 228: 1219–1226. 32729147 10.1111/nph.16822

[nph70882-bib-0027] Chirinos X , Ying S , Rodrigues MA , Maza E , Djari A , Hu G , Liu M , Purgatto E , Fournier S , Regad F *et al*. 2023. Transition to ripening in tomato requires hormone‐controlled genetic reprogramming initiated in gel tissue. Plant Physiology 191: 610–625. 36200876 10.1093/plphys/kiac464 PMC9806557

[nph70882-bib-0028] Colombié S , Beauvoit B , Nazaret C , Bénard C , Vercambre G , Le Gall S , Biais B , Cabasson C , Maucourt M , Bernillon S *et al*. 2017. Respiration climacteric in tomato fruits elucidated by constraint‐based modelling. New Phytologist 213: 1726–1739. 27861943 10.1111/nph.14301 PMC6079640

[nph70882-bib-0029] Colombié S , Prigent S , Cassan C , Hilbert‐Masson G , Renaud C , Dell'Aversana E , Carillo P , Moing A , Beaumont C , Beauvoit B *et al*. 2023. Comparative constraint‐based modelling of fruit development across species highlights nitrogen metabolism in the growth‐defence trade‐off. The Plant Journal 116: 786–803. 37531405 10.1111/tpj.16409

[nph70882-bib-0030] Considine M , Daley DO , Whelan J . 2001. The expression of alternative oxidase and uncoupling protein during fruit ripening in mango. Plant Physiology 126: 1619–1629. 11500560 10.1104/pp.126.4.1619 PMC117161

[nph70882-bib-0031] Contreras C , Tjellström H , Beaudry RM . 2016. Relationships between free and esterified fatty acids and LOX‐derived volatiles during ripening in apple. Postharvest Biology and Technology 112: 105–113.

[nph70882-bib-0032] Costa JH , McDonald AE , Arnholdt‐Schmitt B , de Fernans Melo D . 2014. A classification scheme for alternative oxidases reveals the taxonomic distribution and evolutionary history of the enzyme in angiosperms. Mitochondrion 19: 172–183. 24751423 10.1016/j.mito.2014.04.007

[nph70882-bib-0033] Dal Cin V , Tieman DM , Tohge T , McQuinn R , de Vos RCH , Osorio S , Schmelz EA , Taylor MG , Smits‐Kroon MT , Schuurink RC *et al*. 2011. Identification of genes in the phenylalanine metabolic pathway by ectopic expression of a MYB transcription factor in tomato fruit. Plant Cell 23: 2738–2753. 21750236 10.1105/tpc.111.086975 PMC3226207

[nph70882-bib-0034] Dalla Costa L , Emanuelli F , Trenti M , Moreno‐Sanz P , Lorenzi S , Coller E , Moser S , Slaghenaufi D , Cestaro A , Larcher R *et al*. 2018. Induction of terpene biosynthesis in berries of microvine transformed with VvDXS1 alleles. Frontiers in Plant Science 8: 2244. 29387072 10.3389/fpls.2017.02244 PMC5776104

[nph70882-bib-0035] Daloso DM , Müller K , Obata T , Florian A , Tohge T , Bottcher A , Riondet C , Bariat L , Carrari F , Nunes‐Nesi A *et al*. 2015. Thioredoxin, a master regulator of the tricarboxylic acid cycle in plant mitochondria. Proceedings of the National Academy of Sciences, USA 112: E1392–E1400. 10.1073/pnas.1424840112 PMC4371975 25646482

[nph70882-bib-0036] Decros G , Dussarrat T , Baldet P , Cassan C , Cabasson C , Dieuaide‐Noubhani M , Destailleur A , Flandin A , Prigent S , Mori K *et al*. 2023. Enzyme‐based kinetic modelling of ASC–GSH cycle during tomato fruit development reveals the importance of reducing power and ROS availability. New Phytologist 240: 242–257. 37548068 10.1111/nph.19160

[nph70882-bib-0037] Del‐Saz NF , Ribas‐Carbo M , McDonald AE , Lambers H , Fernie AR , Florez‐Sarasa I . 2018. An *in vivo* perspective of the role s of the alternative oxidase pathway. Trends in Plant Science 23: 206–219. 29269217 10.1016/j.tplants.2017.11.006

[nph70882-bib-0038] Ding Z , Fu L , Wang B , Ye J , Ou W , Yan Y , Li M , Zeng L , Dong X , Tie W *et al*. 2023. Metabolic GWAS‐based dissection of genetic basis underlying nutrient quality variation and domestication of cassava storage root. Genome Biology 24: 289. 38098107 10.1186/s13059-023-03137-y PMC10722858

[nph70882-bib-0172] Eaks IL . 1983. Effects of chilling on respiration and ethylene production of ‘Hass’ avocado fruit at 20 C. Horticultural Science 18: 235–237.

[nph70882-bib-0039] Enfissi EMA , Fraser PD , Lois LM , Boronat A , Schuch W , Bramley PM . 2005. Metabolic engineering of the mevalonate and non‐mevalonate isopentenyl diphosphate‐forming pathways for the production of health‐promoting isoprenoids in tomato. Plant Biotechnology Journal 3: 17–27. 17168896 10.1111/j.1467-7652.2004.00091.x

[nph70882-bib-0040] Etienne A , Génard M , Lobit P , Mbeguié‐A‐Mbéguié D , Bugaud C . 2013. What controls fleshy fruit acidity? A review of malate and citrate accumulation in fruit cells. Journal of Experimental Botany 646: 1451–1469. 10.1093/jxb/ert035 23408829

[nph70882-bib-0173] Fabi JP , Cordenunsi BR , de Mattos Barreto GP , Mercadante AZ , Lajolo FM , Oliveira do Nascimento JR . 2007. Papaya fruit ripening: response to ethylene and 1‐methylcyclopropene (1‐MCP). Journal of Agricultural and Food Chemistry 55: 6118–6123. 17602654 10.1021/jf070903c

[nph70882-bib-0042] Fenech M , Amaya I , Valpuesta V , Botella MA . 2019. Vitamin C content in fruits: biosynthesis and regulation. Frontiers in Plant Science 9: 2006. 30733729 10.3389/fpls.2018.02006 PMC6353827

[nph70882-bib-0043] Flores FB , Martnez‐Madrid MC , Romojaro F . 2008. Influence of fruit development stage on the physiological response to ethylene in cantaloupe charentais melon. Food Science and Technology International 14: 87–94.

[nph70882-bib-0174] Gamrasni D , Feldmesser E , Ben‐Arie R , Raz A , Tabatznik Asiag A , Glikman M , Aharoni A , Goldway M . 2020. Gene expression in 1‐Methylcyclopropene (1‐MCP) treated tomatoes during pre‐climacteric ripening suggests shared regulation of methionine biosynthesis, ethylene production and respiration. Agronomy 10: 1669.

[nph70882-bib-0044] Gane R . 1934. Production of ethylene by some ripening fruits. Nature 134: 1008.

[nph70882-bib-0045] Garrido A , Conde A , Serôdio J , De Vos RCH , Cunha A . 2023. Fruit photosynthesis: more to know about where, how and why. Plants 12: 2393. 37446953 10.3390/plants12132393 PMC10347175

[nph70882-bib-0046] Giordano A , Santo Domingo M , Quadrana L , Pujol M , Martín‐Hernández AM , Garcia‐Mas J . 2022. CRISPR/Cas9 gene editing uncovers the roles of CONSTITUTIVE TRIPLE RESPONSE 1 and REPRESSOR OF SILENCING 1 in melon fruit ripening and epigenetic regulation. Journal of Experimental Botany 73: 4022–4033. 35394503 10.1093/jxb/erac148

[nph70882-bib-0047] Giovannoni J , Nguyen C , Ampofo B , Zhong S , Fei Z . 2017. The epigenome and transcriptional dynamics of fruit ripening. Annual Review of Plant Biology 68: 61–84. 10.1146/annurev-arplant-042916-040906 28226232

[nph70882-bib-0048] Gonda I , Bar E , Davidovich‐Rikanati R , Fait A , Katzir N , Lewinsohn E . 2026. Functional characterization of a ripening‐specific L‐methionine aminotransferase and its role in volatile C3‐thioether esters biosynthesis in melon fruits. Plant Science 363: 112809. 10.1016/j.plantsci.2025.112809 41072806

[nph70882-bib-0049] Gonda I , Bar E , Portnoy V , Lev S , Burger J , Schaffer AA , Tadmor Y , Gepstein S , Giovannoni JJ , Katzir N *et al*. 2010. Branched‐chain and aromatic amino acid catabolism into aroma volatiles in *Cucumis melo* L. fruit. Journal of Experimental Botany 61: 1111–1123. 20065117 10.1093/jxb/erp390 PMC2826658

[nph70882-bib-0050] Gonda I , Burger Y , Schaffer AA . 2016. Biosynthesis and perception of melon aroma. In: Havkin‐Frenkel D , Dudai N , eds. Biotechnology in flavor production, 2^nd^ edn. John Wiley & Sons, Ltd, 281–305.

[nph70882-bib-0051] Gonda I , Davidovich‐Rikanati R , Bar E , Lev S , Jhirad P , Meshulam Y , Wissotsky G , Portnoy V , Burger J , Schaffer AA *et al*. 2018. Differential metabolism of L‐phenylalanine in the formation of aromatic volatiles in melon *Cucumis melo* L. fruit. Phytochemistry 148: 122–131. 29448137 10.1016/j.phytochem.2017.12.018

[nph70882-bib-0052] Goodenough PW , Prosser IM , Young K . 1985. NADP‐linked malic enzyme and malate metabolism in ageing tomato fruit. Phytochemistry 24: 1157–1162.

[nph70882-bib-0053] Guillet C , Just D , Bénard N , Destrac‐Irvine A , Baldet P , Hernould M , Causse M , Raymond P , Rothan C . 2002. A fruit‐specific phosphoenolpyruvate carboxylase is related to rapid growth of tomato fruit. Planta 214: 717–726. 11882940 10.1007/s00425-001-0687-z

[nph70882-bib-0054] Gupta KJ , Kumari A , Florez‐Sarasa I , Fernie AR , Igamberdiev AU . 2018. Interaction of nitric oxide with the components of the plant mitochondrial electron transport chain. Journal of Experimental Botany 69: 3413–3424. 29590433 10.1093/jxb/ery119

[nph70882-bib-0055] Hadfield KA , Rose JK , Bennett AB . 1995. The respiratory climacteric is present in Charentais (*Cucumis melo* cv. Reticulatus F1 Alpha) melons ripened on or off the plant. Journal of Experimental Botany 46: 1923–1925.

[nph70882-bib-0175] Hansen E . 1942. Quantitative study of ethylene production in relation to respiration of pears. Botanical Gazette 103: 543–558.

[nph70882-bib-0056] He C , Liew LC , Yin L , Lewsey MG , Whelan J , Berkowitz O . 2022. The retrograde signaling regulator ANAC017 recruits the MKK9–MPK3/6, ethylene, and auxin signaling pathways to balance mitochondrial dysfunction with growth. Plant Cell 34: 3460–3481. 35708648 10.1093/plcell/koac177 PMC9421482

[nph70882-bib-0176] Herner RC , JrKC S . 1973. Ethylene production and respiratory behavior of the rin tomato mutant. Plant Physiology 52: 38–42. 16658495 10.1104/pp.52.1.38 PMC366434

[nph70882-bib-0057] Hewitt S , Dhingra A . 2020. Beyond ethylene: new insights regarding the role of alternative oxidase in the respiratory climacteric. Frontiers in Plant Science 11: 543958. 33193478 10.3389/fpls.2020.543958 PMC7652990

[nph70882-bib-0058] Holtzapffel RC , Finnegan PM , Millar AH , Badger MR , Day DA . 2002. Mitochondrial protein expression in tomato fruit during on‐vine ripening and cold storage. Functional Plant Biology 29: 827–834. 32689530 10.1071/PP01245

[nph70882-bib-0059] Huang W , Hu N , Xiao Z , Qiu Y , Yang Y , Yang J , Mao X , Wang Y , Li Z , Guo H . 2022. A molecular framework of ethylene‐mediated fruit growth and ripening processes in tomato. Plant Cell 34: 3280–3300. 35604102 10.1093/plcell/koac146 PMC9421474

[nph70882-bib-0060] Hulme AC , Jones JD , Wooltorto LSC . 1963. The respiration climacteric in apple fruits. Proceedings of the Royal Society of London, Series B: Biological Sciences 158: 514–535.

[nph70882-bib-0061] Hunter DA , O'Donnell K , Zhang H , Erridge ZA , Napier NJ , Pidakala P , Baylis E , Saei A , Günther C , Cooney JM *et al*. 2024. On‐tree ripening block of avocado fruit involves changes in ethylene sensitivity linked to gibberellin and auxin pathways. Postharvest Biology and Technology 216: 113031.

[nph70882-bib-0062] Igamberdiev AU , Bykova NV . 2023. Mitochondria in photosynthetic cells: Coordinating redox control and energy balance. Plant Physiology 191: 2104–2119. 36440979 10.1093/plphys/kiac541 PMC10069911

[nph70882-bib-0063] Iglesias‐Sanchez A , Del‐Saz NF , Ezquerro M , Feixes‐Prats E , Ribas‐Carbo M , Fernie AR , Rodríguez‐Concepción M , Florez‐Sarasa I . 2025. Activation of alternative oxidase ensures carbon supply for ethylene and carotenoid biosynthesis during tomato fruit ripening. Plant Physiology 199: kiaf516. 41102481 10.1093/plphys/kiaf516 PMC12596273

[nph70882-bib-0064] Inaba A , Nakamura R . 1988. Numerical expression for estimating the minimum ethylene exposure time necessary to induce ripening in banana fruit. Journal of the American Society for Horticultural Science 113: 561–564.

[nph70882-bib-0065] Jethva J , Lichtenauer S , Schmidt‐Schippers R , Steffen‐Heins A , Poschet G , Wirtz M , van Dongen JT , Eirich J , Finkemeier I , Bilger W *et al*. 2023. Mitochondrial alternative NADH dehydrogenases NDA1 and NDA2 promote survival of reoxygenation stress in Arabidopsis by safeguarding photosynthesis and limiting ROS generation. New Phytologist 238: 96–112. 36464787 10.1111/nph.18657

[nph70882-bib-0066] Johnston JW , Gunaseelan K , Pidakala P , Wang M , Schaffer RJ . 2009. Co‐ordination of early and late ripening events in apples is regulated through differential sensitivities to ethylene. Journal of Experimental Botany 60: 2689–2699. 19429839 10.1093/jxb/erp122 PMC2692014

[nph70882-bib-0177] Kitamura T , Umemoto T , Iwata T , Akazawa T . 1975. Studies on the storage of melon fruits II. Changes of respiration and ethylene production during ripening with reference to cultivars. Journal of the Japanese Society for Horticultural Science 44: 197–203.

[nph70882-bib-0067] Kochevenko A , Klee HJ , Fernie AR , Araújo WL . 2012. Molecular identification of a further branched‐chain aminotransferase 7 (BCAT7) in tomato plants. Journal of Plant Physiology 169: 437–443. 22226341 10.1016/j.jplph.2011.12.002

[nph70882-bib-0178] Kosiyachinda S , Young RE . 1975. Ethylene production in relation to the initiation of respiratory climacteric in fruit. Plant and Cell Physiology 16: 595–602.

[nph70882-bib-0068] Lai D , Zhang K , He Y , Fan Y , Li W , Shi Y , Gao Y , Huang X , He J , Zhao H *et al*. 2024. Multi‐omics identification of a key glycosyl hydrolase gene FtGH1 involved in rutin hydrolysis in Tartary buckwheat (*Fagopyrum tataricum*). Plant Biotechnology Journal 22: 1206–1223. 38062934 10.1111/pbi.14259 PMC11022807

[nph70882-bib-0069] Laties GG . 1982. The cyanide‐resistant, alternative path in higher plant respiration. Annual Review of Plant Biology 33: 519–555.

[nph70882-bib-0070] Laties GG . 1995. Franklin Kidd, Charles West and F.F. Blackman: the start of modern postharvest physiology. Postharvest Biology and Technology 5: 1–10.

[nph70882-bib-0071] Le XH , Millar AH . 2023. The diversity of substrates for plant respiration and how to optimize their use. Plant Physiology 191: 2133–2149. 36573332 10.1093/plphys/kiac599 PMC10069909

[nph70882-bib-0072] Lemaire‐Chamley M , Mounet F , Deborde C , Maucourt M , Jacob D , Moing A . 2019. NMR‐based tissular and developmental metabolomics of tomato fruit. Metabolites 9: 93. 31075946 10.3390/metabo9050093 PMC6571556

[nph70882-bib-0073] Li C , Li Z , Lu B , Shi Y , Xiao S , Dong H , Zhang R , Liu H , Jiao Y , Xu L *et al*. 2025. Large‐scale metabolomic landscape of edible maize reveals convergent changes in metabolite differentiation and facilitates its breeding improvement. Molecular Plant 18: 619–638. 40025737 10.1016/j.molp.2025.02.007

[nph70882-bib-0074] Li D , Zeng S , Dai R , Chen K . 2025. Slow and steady wins the race: the negative regulators of ethylene biosynthesis in horticultural plants. Horticulture Research 12: uhaf108. 40672964 10.1093/hr/uhaf108 PMC12264428

[nph70882-bib-0075] Li S , Chen K , Grierson D . 2018. A critical evaluation of the role of ethylene and MADS transcription factors in the network controlling fleshy fruit ripening. New Phytologist 221: 1724–1741. 30328615 10.1111/nph.15545

[nph70882-bib-0076] Li X , Qi L , Zang N , Zhao L , Sun Y , Huang X , Wang H , Yin Z , Wang A . 2022. Integrated metabolome and transcriptome analysis of the regulatory network of volatile ester formation during fruit ripening in pear. Plant Physiology and Biochemistry 185: 80–90. 35661588 10.1016/j.plaphy.2022.04.030

[nph70882-bib-0077] Li X , Terzoudis K , Hertog MLATM , Nicolaï BM . 2025. Tissue‐specific responses of the central carbon metabolism to low oxygen stress in tomato fruit. Journal of Experimental Botany 76: 6355–6373. doi: 10.1093/jxb/eraf018. 39869006

[nph70882-bib-0078] Li Y , Qi H , Jin Y , Tian X , Sui L , Qiu Y . 2016. Role of ethylene in biosynthetic pathway of related‐aroma volatiles derived from amino acids in oriental sweet melons (*Cucumis melo* var. *makuwa* Makino). Scientia Horticulturae 201: 24–35.

[nph70882-bib-0079] Liu B , Ouyang Z , Zhang Y , Li X , Hong Y , Huang L , Liu S , Zhang H , Li D , Song F . 2014. Tomato NAC transcription factor SlSRN1 positively regulates defense response against biotic stress but negatively regulates abiotic stress response. PLoS ONE 9: e102067. 25010573 10.1371/journal.pone.0102067 PMC4092073

[nph70882-bib-0179] Liu S , Yang Y , Murayama H , Taira S , Fukushima T . 2004. Effects of CO_2_ on respiratory metabolism in ripening banana fruit. Postharvest Biology and Technology 33: 27–34.

[nph70882-bib-0080] Liu B , Santo Domingo M , Mayobre C , Martín‐Hernández AM , Pujol M , Garcia‐Mas J . 2022. Knock‐out of CmNAC‐NOR affects melon climacteric fruit ripening. Frontiers in Plant Science 13: 878037. 35755703 10.3389/fpls.2022.878037 PMC9226586

[nph70882-bib-0081] Liu M , Pirrello J , Chervin C , Roustan JP , Bouzayen M . 2015. Ethylene control of fruit ripening: revisiting the complex network of transcriptional regulation. Plant Physiology 169: 2380–2390. 26511917 10.1104/pp.15.01361 PMC4677914

[nph70882-bib-0082] Liu M , Zeng J , Li T , Li Y , Jiang Y , Duan X , Jiang G . 2024. Transcription factor NOR and CNR synergistically regulate tomato fruit ripening and carotenoid biosynthesis. Molecular Horticulture 4: 27. 38978069 10.1186/s43897-024-00103-5 PMC11232299

[nph70882-bib-0083] Loreto F , Centritto M , Barta C , Calfapietra C , Fares S , Monson RK . 2007. The relationship between isoprene emission rate and dark respiration rate in white poplar (*Populus alba* L.) leaves. Plant, Cell & Environment 30: 662–669. 10.1111/j.1365-3040.2007.01648.x 17407543

[nph70882-bib-0180] Lyons JM , McGlasson WB , Pratt HK . 1962. Ethylene production, respiration, & internal gas concentrations in cantaloupe fruits at various stages of maturity. Plant Physiology 37: 31. 16655605 10.1104/pp.37.1.31 PMC549733

[nph70882-bib-0084] Lytovchenko A , Eickmeier I , Pons C , Osorio S , Szecowka M , Lehmberg K , Arrivault S , Tohge T , Pineda B , Anton MT *et al*. 2011. Tomato fruit photosynthesis is seemingly unimportant in primary metabolism and ripening but plays a considerable role in seed development. Plant Physiology 157: 1650–1663. 21972266 10.1104/pp.111.186874 PMC3327185

[nph70882-bib-0181] Mallick MFR , Masui M , Ishida A , Nukaya A . 1984. Respiration and ethylene production in muskmelons in relation to nitrogen and calcium nutrition. Journal of the Japanese Society for Horticultural Science 52: 429–433.

[nph70882-bib-0085] Manjunatha G , Gupta K , Veeresh Lokesh V , Mur L , Neelwarne B . 2012. Nitric oxide counters ethylene effects on ripening fruits. effects on ripening fruits. Plant Signaling & Behavior 7: 476–483. 22499176 10.4161/psb.19523 PMC3419037

[nph70882-bib-0086] Maoz I , Lewinsohn E , Gonda I . 2022. Amino acids metabolism as a source for aroma volatiles biosynthesis. Current Opinion in Plant Biology 67: 102221. 35533493 10.1016/j.pbi.2022.102221

[nph70882-bib-0182] Martinez G , Serrano M , Pretel MT , Riquelme F , Romojaro F . 1993. Ethylene biosynthesis and physico‐chemical changes during fruit ripening of cherimoya (*Annona cherimola*, Mill). Journal of Horticultural Science 68: 477–483.

[nph70882-bib-0087] Martinez Rivas FJ , Smith M , Zagnishei Z , Alseekh S , Usadel B , Plaxton W , Fernie AR . 2025. Malate matters: disrupting bacterial‐type phosphoenolpyruvate carboxylase (BTPC) rewires tomato fruit development. Plant Physiology doi: 10.1093/plphys/kiag026. PMC13172252 41604419

[nph70882-bib-0088] Matos IF , Morales LMM , Santana DB , Silva GMC , Gomes MMA , Ayub RA , Costa JH , Oliveira JG . 2022. Ascorbate synthesis as an alternative electron source for mitochondrial respiration: possible implications for the plant performance. Frontiers in Plant Science 13: 987077. 36507441 10.3389/fpls.2022.987077 PMC9727407

[nph70882-bib-0089] Mayobre C , Garcia‐Mas J , Pujol M . 2024. A matter of smell: the complex regulation of aroma production in melon. Food Chemistry 460: 140640. 39096801 10.1016/j.foodchem.2024.140640

[nph70882-bib-0090] McDonald AE . 2023. Unique opportunities for future research on the alternative oxidase of plants. Plant Physiology 191: 2084–2092. 36472529 10.1093/plphys/kiac555 PMC10069896

[nph70882-bib-0183] McMurchie EJ , McGlasson WB , Eaks IL . 1972. Treatment of fruit with propylene gives information about the biogenesis of ethylene. Nature 237: 235–236. 4557321 10.1038/237235a0

[nph70882-bib-0091] Merendino L , Courtois F , Grübler B , Bastien O , Straetmanns V , Chevalier F , Lerbs‐Mache S , Lurin C , Pfannschmidt T . 2020. Retrograde signals from mitochondria reprogramme skoto‐morphogenesis in *Arabidopsis thaliana* via alternative oxidase 1a. Philosophical Transactions of the Royal Society B 375: 20190567. 10.1098/rstb.2019.0567 PMC7209964 32362252

[nph70882-bib-0092] Monné M , Daddabbo L , Gagneul D , Obata T , Hielscher B , Palmieri L , Miniero DV , Fernie AR , Weber APM , Palmieri F . 2018. Uncoupling proteins 1 and 2 (UCP1 and UCP2) from *Arabidopsis thaliana* are mitochondrial transporters of aspartate, glutamate, and dicarboxylates. Journal of Biological Chemistry 293: 4213–4227. 29371401 10.1074/jbc.RA117.000771 PMC5857996

[nph70882-bib-0093] Muñoz P , Munné‐Bosch S . 2018. Photo‐oxidative stress during leaf, flower and fruit development. Plant Physiology 176: 1004–1014. 29051197 10.1104/pp.17.01127 PMC5813531

[nph70882-bib-0094] Nashilevitz S , Melamed‐Bessudo C , Izkovich Y , Rogachev I , Osorio S , Itkin M , Adato A , Pankratov I , Hirschberg J , Fernie AR *et al*. 2010. An orange ripening mutant links plastid NADPH dehydrogenase complex activity to central and specialized metabolism during tomato fruit maturation. Plant Cell 22: 1977–1997. 20571113 10.1105/tpc.110.074716 PMC2910969

[nph70882-bib-0525] Nelson RC . 1939. Production and consumption of ethylene by ethylene‐treated bananas. Plant Physiology 14: 817–822. 16653604 10.1104/pp.14.4.817 PMC437790

[nph70882-bib-0184] Nelson RC . 1940. Quantitative study of the production of ethylene by ripening McIntosh apples. Plant Physiology 15: 149–151. 16653618 10.1104/pp.15.1.149 PMC438261

[nph70882-bib-0095] Nicolas P , Shinozaki Y , Powell A , Philippe G , Snyder SI , Bao K , Zheng Y , Xu Y , Courtney L , Vrebalov J *et al*. 2022. Spatiotemporal dynamics of the tomato fruit transcriptome under prolonged water stress. Plant Physiology 1904: 2557–2578. 10.1093/plphys/kiac445 PMC9706477 36135793

[nph70882-bib-0096] Nordey T , Léchaudel M , Génard M , Joas J . 2016. Factors affecting ethylene and carbon dioxide concentrations during ripening: incidence on final dry matter, total soluble solids content and acidity of mango fruit. Journal of Plant Physiology 196: 70–78. 27085177 10.1016/j.jplph.2016.03.008

[nph70882-bib-0097] O'Leary BM , Asao S , Millar AH , Atkin OK . 2019. Core principles which explain variation in respiration across biological scales. New Phytologist 222: 670–686. 30394553 10.1111/nph.15576

[nph70882-bib-0098] Oliveira MG , Mazorra LM , Souza AF , Silva GMC , Correa SF , Santos WC , Saraiva KDC , Jr AJT , Melo DF , Silva MG *et al*. 2015. Involvement of AOX and UCP pathways in the post‐harvest ripening of papaya fruits. Journal of Plant Physiology 189: 42–50. 26513459 10.1016/j.jplph.2015.10.001

[nph70882-bib-0099] Osorio S , Carneiro RT , Lytovchenko A , McQuinn R , Sørensen I , Vallarino JG , Giovannoni JJ , Fernie AR , Rose JKC . 2020. Genetic and metabolic effects of ripening mutations and vine detachment on tomato fruit quality. Plant Biotechnology Journal 18: 106–118. 31131540 10.1111/pbi.13176 PMC6920187

[nph70882-bib-0100] Osorio S , Scossa F , Fernie AR . 2013. Molecular regulation of fruit ripening. Frontiers in Plant Science 4: 198. 23785378 10.3389/fpls.2013.00198 PMC3682129

[nph70882-bib-0101] Pattyn J , Vaughan‐Hirsch J , Van de Poel B . 2021. The regulation of ethylene biosynthesis: a complex multilevel control circuitry. New Phytologist 229: 770–782. 32790878 10.1111/nph.16873 PMC7820975

[nph70882-bib-0102] Paul V , Pandey R , Srivastava GC . 2012. The fading distinctions between classical patterns of ripening in climacteric and non‐climacteric fruit and the ubiquity of ethylene‐an overview. Journal of Food Science and Technology 49: 1–21. 23572821 10.1007/s13197-011-0293-4 PMC3550874

[nph70882-bib-0185] Paull RE . 1982. Postharvest variation in composition of soursop (*Annona muricata* L.) fruit in relation to respiration and ethylene production. Journal of the American Society for Horticultural Science 107: 582–585.

[nph70882-bib-0103] Pech JC , Bouzayen M , Latché A . 2008. Climacteric fruit ripening: Ethylene‐dependent and independent regulation of ripening pathways in melon fruit. Plant Science 175: 114–120.

[nph70882-bib-0104] Pereira L , Pujol M , Garcia‐Mas J , Phillips MA . 2017. Non‐invasive quantification of ethylene in attached fruit headspace at 1 p.p.b. by gas chromatography–mass spectrometry. The Plant Journal 91: 172–183. 28370685 10.1111/tpj.13545

[nph70882-bib-0105] Perez de Souza L , Garbowicz K , Brotman Y , Tohge T , Fernie AR . 2020. The acetate pathway supports flavonoid and lipid biosynthesis in Arabidopsis. Plant Physiology 182: 857–869. 31719153 10.1104/pp.19.00683 PMC6997690

[nph70882-bib-0106] Perotti MF , Posé D , Martín‐Pizarro C . 2023. Non‐climacteric fruit development and ripening regulation: ‘the phytohormones show’. Journal of Experimental Botany 74: 6237–6253. 37449770 10.1093/jxb/erad271 PMC10627154

[nph70882-bib-0107] Perotti VE , Moreno AS , Podestá FE . 2014. Physiological aspects of fruit ripening: The mitochondrial connection. Mitochondrion 17: 1–6. 24769052 10.1016/j.mito.2014.04.010

[nph70882-bib-0108] Pott DM , Osorio S , Vallarino JG . 2019. From central to specialized metabolism: AN overview of some secondary compounds derived from the primary metabolism for their role in conferring nutritional and organoleptic characteristics to fruit. Frontiers in Plant Science 10: 835–854. 31316537 10.3389/fpls.2019.00835 PMC6609884

[nph70882-bib-0109] Pujol M , Garcia‐Mas J . 2023. Regulation of climacteric fruit ripening in melon: recent advances and future challenges. Journal of Experimental Botany 74: 6224–6236. 37399085 10.1093/jxb/erad256

[nph70882-bib-0110] Qin G , Tao S , Zhang H , Huang W , Wu J , Xu Y , Zhang S . 2014. Evolution of the aroma volatiles of pear fruits supplemented with fatty acid metabolic precursors. Molecules 19: 20183–20196. 25474290 10.3390/molecules191220183 PMC6271835

[nph70882-bib-0111] Rasmusson AG , Escobar MA , Hao M , Podgórska A , Szal B . 2020. Mitochondrial NADPH oxidation pathways and nitrate/ammonium redox balancing in plants. Mitochondrion 53: 158–165. 32485334 10.1016/j.mito.2020.05.010

[nph70882-bib-0112] Renato M , Pateraki I , Boronat A , Azcón‐Bieto J . 2014. Tomato fruit chromoplasts behave as respiratory bioenergetic organelles during ripening. Plant Physiology 166: 920–933. 25125503 10.1104/pp.114.243931 PMC4213118

[nph70882-bib-0186] Resende ECO , Martins PF , Azevedo RAD , Jacomino AP , Bron IU . 2012. Oxidative processes during ‘Golden’ papaya fruit ripening. Brazilian Journal of Plant Physiology 24: 85–94.

[nph70882-bib-0113] Ríos P , Argyris J , Vegas J , Leida C , Kenigswald M , Tzuri G , Troadec C , Bendahmane A , Katzir N , Picó B *et al*. 2017. ETHQV6.3 is involved in melon climacteric fruit ripening and is encoded by a NAC domain transcription factor. The Plant Journal 91: 671–683. 28493311 10.1111/tpj.13596

[nph70882-bib-0114] Roch L , Prigent S , Klose H , Cakpo CB , Beauvoit B , Deborde C , Fouillen P , van Delft P , Jacob D , Usadel B *et al*. 2020. Biomass composition explains fruit relative growth rate and discriminates climacteric from non‐climacteric species. Journal of Experimental Botany 71: 5823–5836. 32592486 10.1093/jxb/eraa302 PMC7540837

[nph70882-bib-0115] Rodriguez‐Concepcion M , Avalos J , Bonet ML , Boronat A , Gomez‐Gomez L , Hornero‐Mendez D , Limon MC , Meléndez‐Martínez AJ , Olmedilla‐Alonso B , Palou A *et al*. 2018. A global perspective on carotenoids: metabolism, biotechnology, and benefits for nutrition and health. Progress in Lipid Research 70: 62–93. 29679619 10.1016/j.plipres.2018.04.004

[nph70882-bib-0116] Rogiers S , Knowles R . 1999. A comparison of preharvest and postharvest ethylene production and respiration rates of saskatoon (*Amelanchier alnifolia* Nutt.) fruit during development. Canadian Journal of Botany 77: 323–332.

[nph70882-bib-0187] Rudell DR , Mattinson DS , Fellman JK , Mattheis JP . 2000. The progression of ethylene production and respiration in the tissues of ripening ‘Fuji’ apple fruit. Horticultural Science 35: 1300–1303.

[nph70882-bib-0117] Sadali NM , Sowden RG , Ling Q , Jarvis RP . 2019. Differentiation of chromoplasts and other plastids in plants. Plant Cell Reports 38: 803–818. 31079194 10.1007/s00299-019-02420-2 PMC6584231

[nph70882-bib-0118] Saito K , Yonekura‐Sakakibara K , Nakabayashi R , Higashi Y , Yamazaki M , Tohge T , Fernie AR . 2013. The flavonoid biosynthetic pathway in Arabidopsis: structural and genetic diversity. Plant Physiology and Biochemistry 72: 21–34. 23473981 10.1016/j.plaphy.2013.02.001

[nph70882-bib-0119] Saladié M , Wright LP , Garcia‐Mas J , Rodriguez‐Concepcion M , Phillips MA . 2014. The 2‐C‐methylerythritol 4‐phosphate pathway in melon is regulated by specialized isoforms for the first and last steps. Journal of Experimental Botany 6517: 5077–5092. 10.1093/jxb/eru275 PMC4144782 25013119

[nph70882-bib-0120] Saltveit ME Jr . 1993. Internal carbon dioxide and ethylene levels in ripening tomato fruit attached to or detached from the plant. Physiologia Plantarum 89: 204–210.

[nph70882-bib-0188] Sams CE , Conway WS . 1984. Effect of calcium infiltration on ethylene production, respiration rate, soluble polyuronide content, and quality of ‘Golden Delicious’ apple fruit. Journal of the American Society for Horticultural Science 109: 53–57.

[nph70882-bib-0121] Santo Domingo M , Orduña L , Navarro D , Mayobre C , Santiago A , Valverde L , Alexiou KG , Matus JT , Pujol M , Garcia‐Mas J . 2024. The ethylene‐responsive transcription factor ERF024 is a novel regulator of climacteric fruit ripening in melon. The Plant Journal 119: 1844–1858. 38900073 10.1111/tpj.16889

[nph70882-bib-0122] Sawamura M , Knegt E , Bruinsma J . 1978. Levels of endogenous ethylene, carbon dioxide, and soluble pectin, and activities of pectin methylesterase and polygalacturonase in ripening tomato fruits. Plant & Cell Physiology 19: 1061–1069.

[nph70882-bib-0123] Selinski J , Hartmann A , Deckers‐Hebestreit G , Day DA , Whelan J , Scheibe R . 2018. Alternative oxidase isoforms are differentially activated by tricarboxylic acid cycle intermediates. Plant Physiology 176: 1423–1432. 29208641 10.1104/pp.17.01331 PMC5813554

[nph70882-bib-0159] Selinski J , Frings S , Schmidt‐Schippers R . 2024. Perception and processing of stress signals by plant mitochondria. The Plant Journal 120: 2337–2355. doi: 10.1111/tpj.17133. 39527570 PMC11658195

[nph70882-bib-0124] Seymour GB , Østergaard L , Chapman NH , Knapp S , Martin C . 2013. Fruit development and ripening. Annual Review of Plant Biology 64: 219–241. 10.1146/annurev-arplant-050312-120057 23394500

[nph70882-bib-0125] Sfakiotakis EM , Dilley DR . 1973. Internal ethylene concentrations in apple fruits attached to or detached from the tree. Journal of the American Society for Horticultural Science 98: 501–503.

[nph70882-bib-0126] Shan N , Zhang Y , Guo Y , Zhang W , Nie J , Fernie AR , Sui X . 2023. Cucumber malate decarboxylase, CsNADP‐ME2, functions in the balance of carbon and amino acid metabolism in fruit. Horticulture Research 10: uhad216. 38077499 10.1093/hr/uhad216 PMC10699846

[nph70882-bib-0127] Sharma E , Anand G , Kapoor R . 2017. Terpenoids in plant and arbuscular mycorrhiza‐reinforced defence against herbivorous insects. Annals of Botany 119: 791–801. 28087662 10.1093/aob/mcw263 PMC5378189

[nph70882-bib-0128] Shellie KC , Saltveit JME . 1993. The lack of a respiratory rise in muskmelon fruit ripening on the plant challenges the definition of climacteric behaviour. Journal of Experimental Botany 44: 1403–1406.

[nph70882-bib-0129] Shiomi S , Wamocho LS , Agong SG . 1996. Ripening characteristics of purple passion fruit on and off the vine. Postharvest Biology and Technology 7: 161–170.

[nph70882-bib-0130] Silva GMC , Morales LMM , Santana DS , Santa‐Catarina C , Oliveira JG . 2024. The alternative respiration is linked with the ascorbate synthesis capacity in climacteric and non‐climacteric fruit mitochondria. Postharvest Biology and Technology 210: 112780.

[nph70882-bib-0131] Simkin AJ , Faralli M , Ramamoorthy S , Lawson T . 2020. Photosynthesis in non‐foliar tissues: implications for yield. The Plant Journal 101: 1001–1015. 31802560 10.1111/tpj.14633 PMC7064926

[nph70882-bib-0189] Solomos T , Laties GG . 1976. Effects of cyanide and ethylene on the respiration of cyani de‐sensitive and cyanide‐resistant plant tissues. Plant Physiology 58: 47–50. 16659618 10.1104/pp.58.1.47 PMC542177

[nph70882-bib-0190] Song J , Bangerth F . 1996. The effect of harvest date on aroma compound production from ‘Golden Delicious’ apple fruit and relationship to respiration and ethylene production. Postharvest Biology and Technology 8: 259–269.

[nph70882-bib-0132] Song J , Bangerth F . 2003. Fatty acids as precursors for aroma volatile biosynthesis in pre‐climacteric and climacteric apple fruit. Postharvest Biology and Technology 30: 113–121.

[nph70882-bib-0191] Spencer MS . 1956. Ethylene metabolism in tomato fruit: I. Relationship of ethylene evolution to fruit respiration and ripening. Canadian Journal of Biochemistry and Physiology 34: 1261–1270. 13374589

[nph70882-bib-0133] Sui X , Shan N , Hu L , Zhang C , Yu C , Ren H , Turgeon R , Zhang Z . 2017. The complex character of photosynthesis in cucumber fruit. Journal of Experimental Botany 68: 1625–1637. 28369547 10.1093/jxb/erx034 PMC5441898

[nph70882-bib-0134] Sweetlove LJ , Lytovchenko A , Morgan M , Nunes‐Nesi A , Taylor NL , Baxter CJ , Eickmeier I , Fernie AR . 2006. Mitochondrial uncoupling protein is required for efficient photosynthesis. Proceedings of the National Academy of Sciences, USA 103: 19587–19592. 10.1073/pnas.0607751103 PMC1748269 17148605

[nph70882-bib-0135] Sweetlove LJ , Ratcliffe RG , Fernie AR . 2025. Non‐canonical plant metabolism. Nature Plants 11: 696–708. 40164785 10.1038/s41477-025-01965-3

[nph70882-bib-0136] Sweetlove LJ , Williams TCR , Cheung CYM , Ratcliffe RG . 2013. Modelling metabolic CO_2_ evolution – a fresh perspective on respiration. Plant, Cell & Environment 36: 1631–1640. 10.1111/pce.12105 23531106

[nph70882-bib-0137] Sweetman C , Deluc LG , Cramer GR , Ford CM , Soole KL . 2009. Regulation of malate metabolism in grape berry and other developing fruits. Phytochemistry 70: 1329–1344. 19762054 10.1016/j.phytochem.2009.08.006

[nph70882-bib-0138] Tang X , Miszta PK , Nazaroff WW , Goldstein AH . 2015. Siloxanes are the most abundant volatile organic compound emitted from engineering students in a classroom. Environmental Science & Technology Letters 2: 303–307.

[nph70882-bib-0139] Terry LI , Roemer RB , Booth DT , Moore CJ , Walter GH . 2016. Thermogenic respiratory processes drive the exponential increase of volatile organic compound emissions in *Macrozamia cycad* cones. Plant, Cell & Environment 39: 1588–1600. 10.1111/pce.12730 26924274

[nph70882-bib-0140] Ties P , Barringer S . 2012. Influence of lipid content and lipoxygenase on flavor volatiles in the tomato peel and flesh. Journal of Food Science 77: C830–C837. 22757705 10.1111/j.1750-3841.2012.02775.x

[nph70882-bib-0141] Tohge T , Zhang Y , Peterek S , Matros A , Rallapalli G , Tandrón YA , Butelli E , Kallam K , Hertkorn N , Mock HP *et al*. 2015. Ectopic expression of snapdragon transcription factors facilitates the identification of genes encoding enzymes of anthocyanin decoration in tomato. The Plant Journal 83: 686–704. 26108615 10.1111/tpj.12920

[nph70882-bib-0192] Trinchero GD , Sozzi GO , Cerri AM , Vilella F , Fraschina AA . 1999. Ripening‐related changes in ethylene production, respiration rate and cell‐wall enzyme activity in goldenberry (*Physalis peruviana* L.), a solanaceous species. Postharvest Biology and Technology 16: 139–145.

[nph70882-bib-0142] Tucker GA . 1993. Biochemistry of fruit ripening. London, UK: Chapman & Hall.

[nph70882-bib-0193] Tucker ML , Laties GG . 1984. Interrelationship of gene expression, polysome prevalence, and respiration during ripening of ethylene and/or cyanide‐treated avocado fruit. Plant Physiology 74: 307–315. 16663414 10.1104/pp.74.2.307 PMC1066674

[nph70882-bib-0143] Upadhyay RK , Mattoo AK . 2018. Genome‐wide identification of tomato (*Solanum lycopersicum* L.) lipoxygenases coupled with expression profiles during plant development and in response to methyl‐jasmonate and wounding. Journal of Plant Physiology 231: 318–328. 30368230 10.1016/j.jplph.2018.10.001

[nph70882-bib-0144] Vanlerberghe GC , Dahal K , Alber NA , Chadee A . 2020. Photosynthesis, respiration and growth: a carbon and energy balancing act for alternative oxidase. Mitochondrion 52: 197–211. 32278748 10.1016/j.mito.2020.04.001

[nph70882-bib-0145] Vogt J , Schiller D , Ulrich D , Schwab W , Dunemann F . 2013. Identification of lipoxygenase LOX genes putatively involved in fruit flavour formation in apple (*Malus × domestica*). Tree Genetics & Genomes 9: 1493–1511.

[nph70882-bib-0146] Walker RP , Chen ZH , Famiani F . 2021. Gluconeogenesis in plants: a key interface between organic acid/amino acid/lipid and sugar metabolism. Molecules 26: 5129. 34500562 10.3390/molecules26175129 PMC8434439

[nph70882-bib-0147] Walker RP , Famiani F . 2018. Organic acids in fruits: metabolism, functions and contents. Horticultural Reviews 45: 371–430.

[nph70882-bib-0194] Wang CY , Mellenthin WM . 1972. Internal ethylene levels during ripening and climacteric in Anjou pears. Plant Physiology 50: 311–312. 16658164 10.1104/pp.50.2.311 PMC366132

[nph70882-bib-0195] Wang YW , Acharya TP , Malladi A , Tsai HJ , NeSmith DS , Doyle JW , Nambeesan SU . 2022. Atypical climacteric and functional ethylene metabolism and signaling during fruit ripening in blueberry (*Vaccinium* sp.). Frontiers in Plant Science 13: 932642. 35812961 10.3389/fpls.2022.932642 PMC9260287

[nph70882-bib-0148] Xu F , Peng Y , He ZQ , Yu LL . 2023. The role of cyanoalanine synthase and alternative oxidase in promoting salt stress tolerance in *Arabidopsis thaliana* . BMC Plant Biology 23: 163. 36973660 10.1186/s12870-023-04167-1 PMC10041793

[nph70882-bib-0149] Xu F , Yuan S , Zhang DW , Lv X , Lin HH . 2012. The role of alternative oxidase in tomato fruit ripening and its regulatory interaction with ethylene. Journal of Experimental Botany 63: 5705–5716. 22915749 10.1093/jxb/ers226 PMC3444281

[nph70882-bib-0150] Xu J , Zhang S . 2015. Ethylene biosynthesis and regulation in plants. In: Wen CK , ed. Ethylene in plants. Dordrecht, the Netherlands: Springer Netherlands, 1–25. doi: 10.1007/978-94-017-9484-8_1.

[nph70882-bib-0151] Zafari S , Vanlerberghe GC , Igamberdiev AU . 2022. The Role of alternative oxidase in the interplay between nitric oxide, reactive oxygen species, and ethylene in tobacco (*Nicotiana tabacum* L.) plants incubated under normoxic and hypoxic conditions. International Journal of Molecular Sciences 23: 7153. 35806157 10.3390/ijms23137153 PMC9266549

[nph70882-bib-0152] Zenoni S , Savoi S , Busatto N , Tornielli GB , Costa F . 2023. Molecular regulation of apple and grape ripening: exploring common and distinct transcriptional aspects of representative climacteric and non‐climacteric fruits. Journal of Experimental Botany 74: 6207–6223. 37591311 10.1093/jxb/erad324 PMC10627160

[nph70882-bib-0153] Zhang B , Yin XR , Li X , Yang SL , Ferguson IB , Chen KS . 2009. Lipoxygenase gene expression in ripening kiwifruit in relation to ethylene and aroma production. Journal of Agricultural and Food Chemistry 57: 2875–2881. 19334761 10.1021/jf9000378

[nph70882-bib-0154] Zhang C , Jin Y , Liu J , Tang Y , Cao S , Qi H . 2014. The phylogeny and expression profiles of the lipoxygenase LOX family genes in the melon *Cucumis melo* L. genome. Scientia Horticulturae 170: 94–102.

[nph70882-bib-0155] Zhang Y , Butelli E , Alseekh S , Tohge T , Rallapalli G , Luo J , Kawar PG , Hill L , Santino A , Fernie AR *et al*. 2015. Multi‐level engineering facilitates the production of phenylpropanoid compounds in tomato. Nature Communications 6: 8635. 10.1038/ncomms9635 PMC4639801 26497596

[nph70882-bib-0156] Zhang Y , Fernie AR . 2023. The role of TCA cycle enzymes in plants. Advanced Biology 7: e2200238. 37341441 10.1002/adbi.202200238

[nph70882-bib-0157] Zheng K , Martinez MDP , Bouzid M , Balparda M , Schwarzländer M , Maurino VG . 2025. Regulation of plant glycolysis and the tricarboxylic acid cycle by posttranslational modifications. The Plant Journal 122: e70142. 40185637 10.1111/tpj.70142 PMC11971034

[nph70882-bib-0158] Zhu F , Wen W , Cheng Y , Fernie AR . 2022. The metabolic changes that effect fruit quality during tomato fruit ripening. Molecular Horticulture 2: 2. 37789428 10.1186/s43897-022-00024-1 PMC10515270

